# Human gut microbe co-cultures have greater potential than monocultures for food waste remediation to commodity chemicals

**DOI:** 10.1038/s41598-018-33733-z

**Published:** 2018-10-22

**Authors:** Matthew A. Perisin, Christian J. Sund

**Affiliations:** US Army Research Laboratory, RDRL-SEE-B, 2800 Powder Mill Road, Adelphi, MD 20783 USA

## Abstract

Food waste represents an underutilized resource for commodity chemical generation. Constituents of the human gut microbiota that are already adapted to a food waste stream could be repurposed for useful chemical production. Industrial fermentations utilizing these microbes maintain organisms in isolation; however, microbial consortia offer an attractive alternative to monocultures in that metabolic interactions may result in more efficient processes with higher yields. Here we computationally assess the ability of co-cultures vs. monocultures to anaerobically convert a Western diet to commodity chemicals. The combination of genome-scale metabolic models with flux-balance analysis predicts that every organism analyzed can benefit from interactions with another microbe, as evidenced by increased biomass fluxes in co-culture vs. monoculture. Furthermore, microbe combinations result in emergent or increased commodity chemical production including butanol, methane, formaldehyde, propionate, hydrogen gas, and urea. These overproducing co-cultures are enriched for mutualistic and commensal interactions. Using *Clostridium beijerinckii* co-cultures as representative examples, models predict cross-fed metabolites will simultaneously modify multiple internal pathways, evident by different internal metabolic network structures. Differences in degree and betweenness centrality of hub precursor metabolites were correlated to *C*. *beijerinckii* metabolic outputs, and thus demonstrate the potential of co-cultures to differentially direct metabolisms to useful products.

## Introduction

Food waste generation in the United States is a widespread remediation problem. A recent study estimated that 0.615 pounds of food waste was produced per person per day^1^. The U.S. Army alone generates tons of food waste each day at contingency operating bases^2^. Standard remediation practices involve combustion and disposal in landfills, thereby perhaps wasting energy dense substrates and generating pollution. Though no longer palatable to humans, food waste is a rich nutrient source for microbial growth. Exposed to the environment, microbes will colonize the waste in diverse consortia, as observed in composting^3–5^. This process will be a complex interplay of microbes cooperating and competing and not just monoculture growth that we are accustomed to in the laboratory. If we can understand the assembly of microbial consortia, then we may be able to direct complex microbial metabolisms to not only efficiently degrade waste streams, but also to direct the products toward commodity chemicals.

Though environmental microbes can degrade food waste, we need not look farther than our own digestive tracts to find a diverse suite of microbes and metabolisms (collectively known as the gut microbiota) that aid digestion of our food. The majority of research has centered on health effects, but these microbes could be used in a new context: food waste remediation to commodity chemicals. Gut microbiota species encode powerful degradative enzymes such as cellulases, hemicellulases, pectinases, and amylases; and produce a plethora of useful compounds including short-chain fatty acids (SCFAs), alcohols, H_2_, CH_4_, polymer precursors, and fertilizers^6,7^. The combinatorial potential of gut microbes’ collective metabolic action is even greater and has remained unexplored for commodity chemical production.

Consortia are advantageous because complex tasks are divided amongst members, leading to reduction of intermediates^8,9^. A diversity in metabolisms also results in high niche occupancy, thus reducing the chance of invasion^10^. There are natural examples of the efficacy of microbial consortia for accomplishing tasks, including fecal microbiota transplants to resolve recurrent *Clostridium difficile* infections^11^ and microbial communities to treat wastewater^12^. Consortia can also work usefully *in vitro*: electrosynthesis of methane, acetate, and hydrogen gas provides an excellent example^13^.

The use of microbial consortia for chemical production is still rare due to difficulty in prediction, and thus control, of community assembly. Furthermore, the number of combinations of species from which to build a consortia is near infinite. A predictive framework is desired to screen and choose members and combinations to produce useful chemicals. Genome-scale metabolic models (GSMMs), combined with flux balance analysis (FBA), provides an attractive framework. To create a GSMM, an annotated genome is the only requirement, which makes this framework advantageous for organisms for which we do not have cultured isolates^14^. GSMMs and FBA have proven remarkably effective in recapitulating single organism metabolisms *in vitro*^15–17^. This type of framework has also been broadened to model organism interactions within consortia under steady-state and dynamic conditions^18–22^. These multi-species metabolic modeling methods allow for computational evaluation of ecological interactions and production potential of microbial consortia, which can inform experimentation.

Metabolic modeling of consortia has suggested different ecological strategies of assembly of natural microbial communities. Levy and Borenstein^23^ used GSMMs to determine seed metabolites (compounds that cannot be endogenously produced) for gut microbes, then compared the overlap of these compounds for co-occurring species in gut microbiome surveys^23,24^. Based on this analysis, co-occurring species generally exhibited a high competition index indicating that habitat filtering is likely to be the major mechanism that structures gut microbiotas^23^. Zelezniak *et al*.^25^ also observed this phenomenon, but further identified subcommittees that displayed a high degree of cross-feeding and cooperativity. Klitgord and Segre^26^ further explored ecological interactions’ reliance on nutrients by computationally finding media components that could push pairwise interactions to be mutualistic. Mutualistic interactions generally involved cross-feeding a growth promoting nutrient or removal of an inhibitory metabolite^26^. Though these studies have broadly informed consortia design, they did not assess the abilities and efficiencies of these communities to produce compounds of interest.

There still remains the question of whether microbial consortia provide additional advantages over monocultures for commodity metabolite production. Chiu *et al*.^27^ computationally identified that pairwise growth of gut microbes can result in emergent metabolite secretion (metabolites not produced by either member in monoculture). Building on these findings, algorithms have been developed to design non-natural consortia for conversion of user specified inputs to targets^28,29^. In particular, the CoMiDA algorithm not only identifies the possible combinatorial paths from input to output, but also minimizes the number species included in that path^28^. However, if one species can produce the output in monoculture, the program will not return other consortia options that may be more efficient or generate higher yields.

Given this challenge, we sought to computationally assess pairs of microbes for ability to not only produce emergent metabolites, but also overproduce compounds of interest. For this screen, we leveraged a large bank (773) of gut microbe GSMMs graciously made available by Magnusdottir *et al*.^30^. These models have all been equally refined and curated to predict anaerobic growth in media based on a Western diet, which we use as a proxy for food waste. As in Magnusdottir *et al*.^30^, we simulated all monocultures and co-cultures, but here we focus on co-cultures that produce commodity chemicals and dissect the metabolic and ecological interactions that contribute to these phenotypes. We find that for every isolate model, there exists a co-culture that increases its biomass production. Co-cultures can produce emergent metabolites, as observed before^27^, and also commodity chemicals in greater than additive ways compared to monocultures. These overproducing co-cultures are enriched for mutualistic and commensal interactions. The number of cross-fed metabolites is also higher for these co-cultures and network analysis indicates that these metabolites simultaneously modify multiple pathways in the internal metabolic networks of each member. Overall, this screen provides the first step towards repurposing human gut microbes to remediate food waste to useful chemicals.

## Results

In this study, we sought to computationally screen microbial consortia for anaerobic remediation of food waste to commodity chemicals. We modeled interactions between microbes with GSMMs combined with FBA. We leveraged research from the human gut microbiome, which has the most sequenced genomes by far from any environment. Recently, Magnusdottir *et al*.^30^ built and refined GSMMs for 773 different gut microbe isolates and kindly made them publicly available. We sought to computationally build consortia with these models, and to make the simplest first step: going from one organism to two. As a proxy for food waste, we used the “Western diet” outlined in Magnusdottir *et al*.^30^, combined with FBA simulations of every pairwise combination of the AGORA models (297549 total simulations) to generate a rich dataset. We asked whether we could re-purpose human gut microbes for commodity chemical production and whether co-culturing could boost production of useful chemicals.

### Comparison to previous results

We first confirmed that we could re-capitulate the results from Magnusdottir *et al*.^30^ using slightly different methods. Instead of the COBRA^31^ Toolbox in MATLAB, we used the *sybil*^32^ package in R. We used the publicly available GLPK solver instead of CPLEX (IBM, Inc.). Finally, we have implemented the AGORA v1.01 model developers’ recent reaction constraint modifications that differ with the published conditions. Given these discrepancies, we still observed good correlation of monoculture biomass fluxes with the anaerobic Western diet (Fig. S1a, Pearson = 0.98, *p* < 2.2e-16). The correlation of co-culture biomass fluxes with the same diet was lower (Fig. S1b,c, Pearson = 0.83, *p* < 2.2e-16).

### Co-culturing to increase biomass production

**Figure 1:**
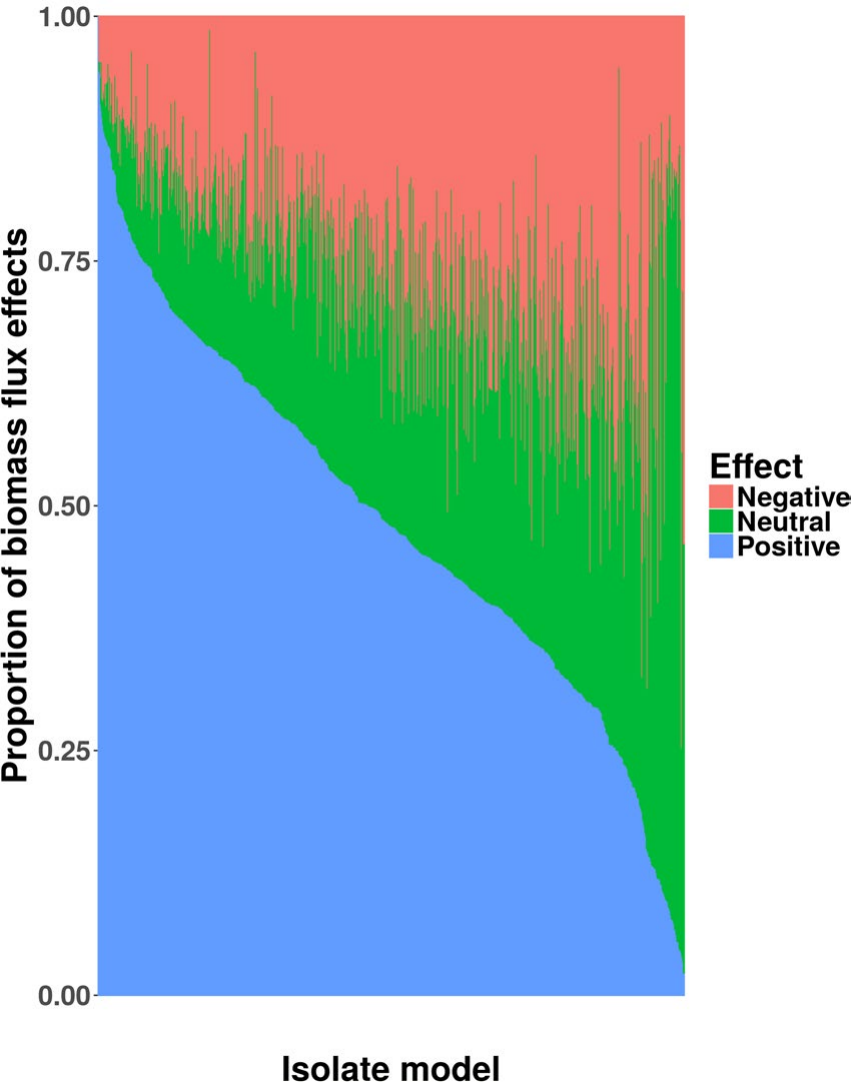
Co-culture FBA simulations predict combinations that increase biomass flux compared to monoculture. For each GSMM (773 total bars along the x-axis), FBA was used to simulate steady-state growth (anaerobic Western diet) of all pairwise combinations of models. The co-culture biomass fluxes were compared to the monoculture biomass flux for each model and deemed positive if the flux was at least 10% greater, negative if the flux was at least 10% less, and neutral otherwise.

To gain insights into overall production of co-cultures, we assessed biomass flux effects in monoculture versus co-culture. For each microbe model, we observed increased biomass production when simulated in the presence of another microbe (Fig. 1). We classified biomass effects as in Magnusdottir *et al*.^30^ (see Methods). We further classified the interaction type for each co-culture (see Methods) and counted the types for all co-culture simulations for each microbe (Supplementary Table 4). This table is useful for selection of strains that are likely to be mutualistic. The model with the most mutualistic co-culture simulations was *Enterococcus saccharolyticus* DSM 8903, followed closely by *Eubacterium desmolans* ATCC 43058 (Supplementary Table 4). These results indicate that co-culturing can be an effective method to boost a microbial biomass production. Furthermore, predicting mutualistic co-cultures is useful to guide future experimental screening of stable, robust consortia.

### Simulations predict the whole is greater than the sum of the parts

**Figure 2:**
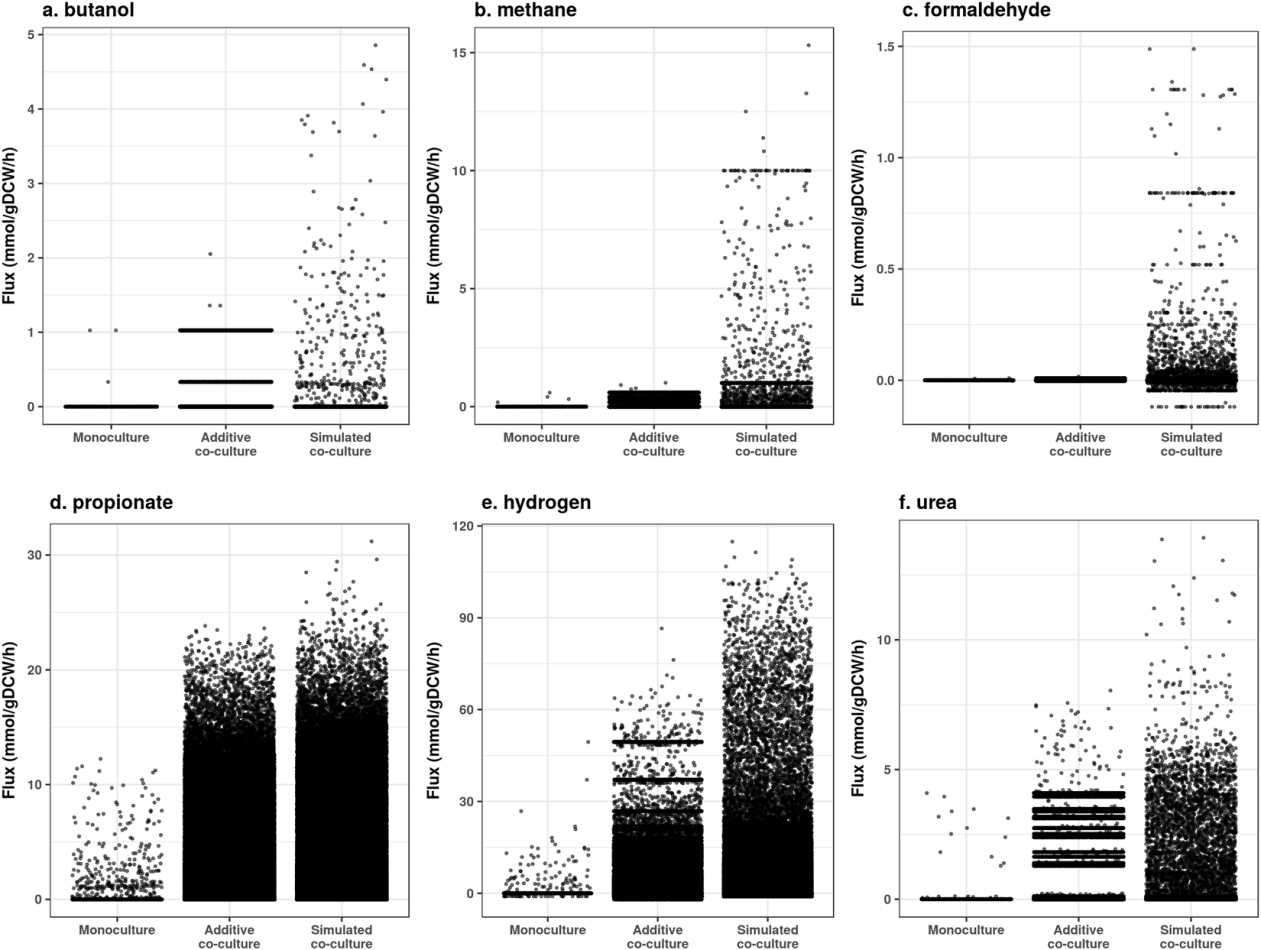
Pairwise simulations predict greater than additive commodity metabolic fluxes. For all monoculture simulations with an anaerobic Western diet, exchange metabolite fluxes were compared to pairwise additions of monoculture fluxes, and to all pairwise co-culture simulations. Commodity metabolites include butanol (**a**), methane (**b**), formaldehyde (**c**), propionate (**d**), hydrogen gas (**e**), and urea (**f**). All flux units are mmol/gDCW/h.

**Figure 3:**
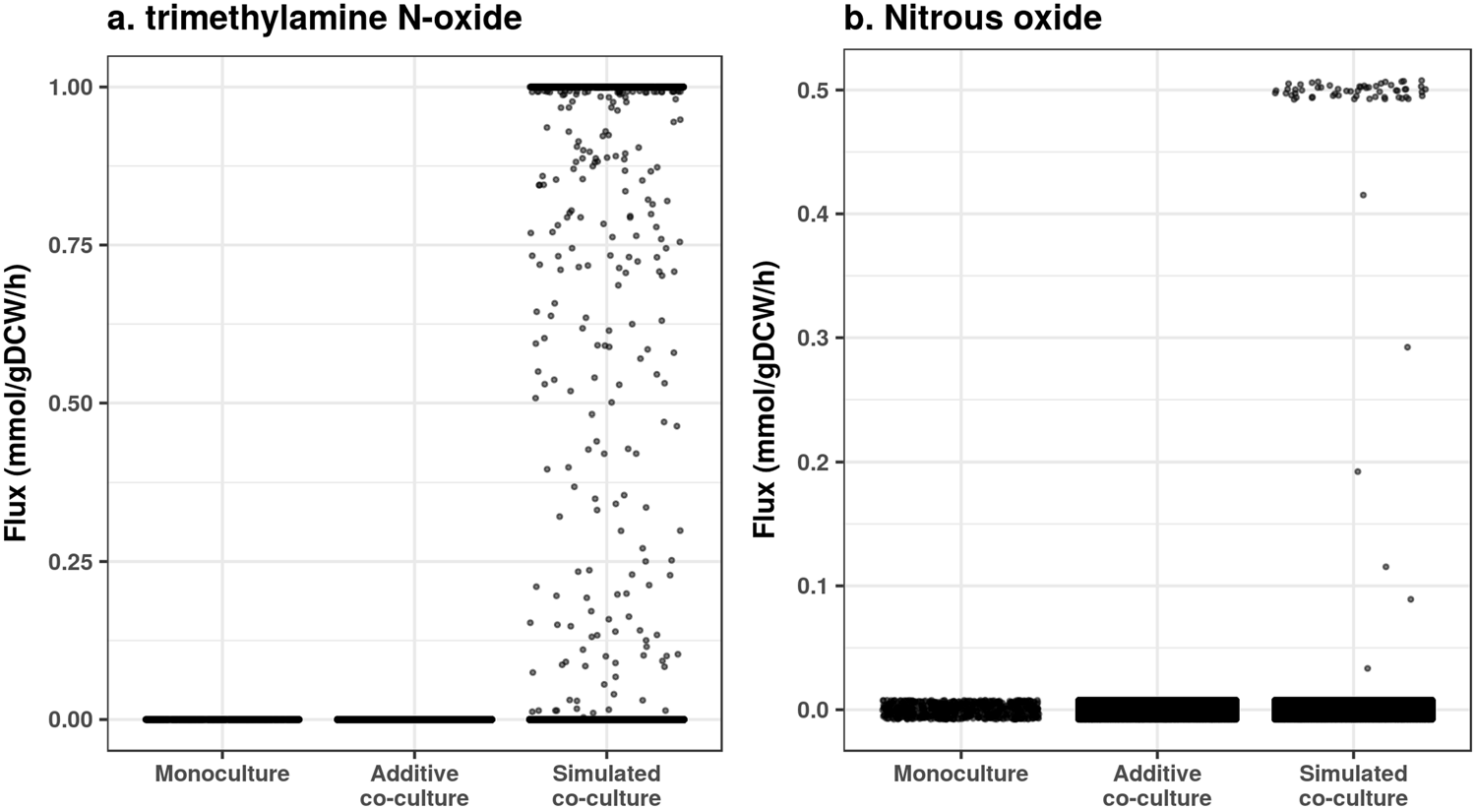
Pairwise simulations predict emergent metabolite fluxes. For all monoculture simulations with an anaerobic Western diet, exchange metabolite fluxes were compared to pairwise additions of monoculture fluxes, and to all pairwise co-culture simulations. Emergent metabolites include trimethylamine N-oxide (**a**) and nitrous oxide (**b**). All flux units are mmol/gDCW/h.

Next, we identified commodity chemicals predicted to be produced by monocultures and asked whether chemical production could be enhanced by co-culturing. We chose chemicals of different classes: butanol (energy dense alcohol), methane and hydrogen (combustible gases), urea (nitrogen fertilizer), propionate (SCFA and polymer precursor), and formaldehyde (resin precursor). We compared export chemical fluxes for all monocultures, all theoretical additive monoculture fluxes, and the simulated co-culture fluxes (Fig. 2), and observed that simulated co-cultures could produce the target chemicals in greater than additive ways (Fig. 2). We refer to this phenotype as the whole is greater than the sum of the parts. Additionally, we observed co-cultures that were predicted to make chemicals not made by either monoculture (emergent metabolites). Some emergent metabolites of interest include trimethylamine N-oxide (TMAO) and nitrous oxide (N_2_O) (Fig. 3). Therefore, co-culturing has the potential to not only boost metabolic production in more than additive ways, but also to extend metabolic capabilities.

### Characteristics of overproducing co-cultures

**Figure 4:**
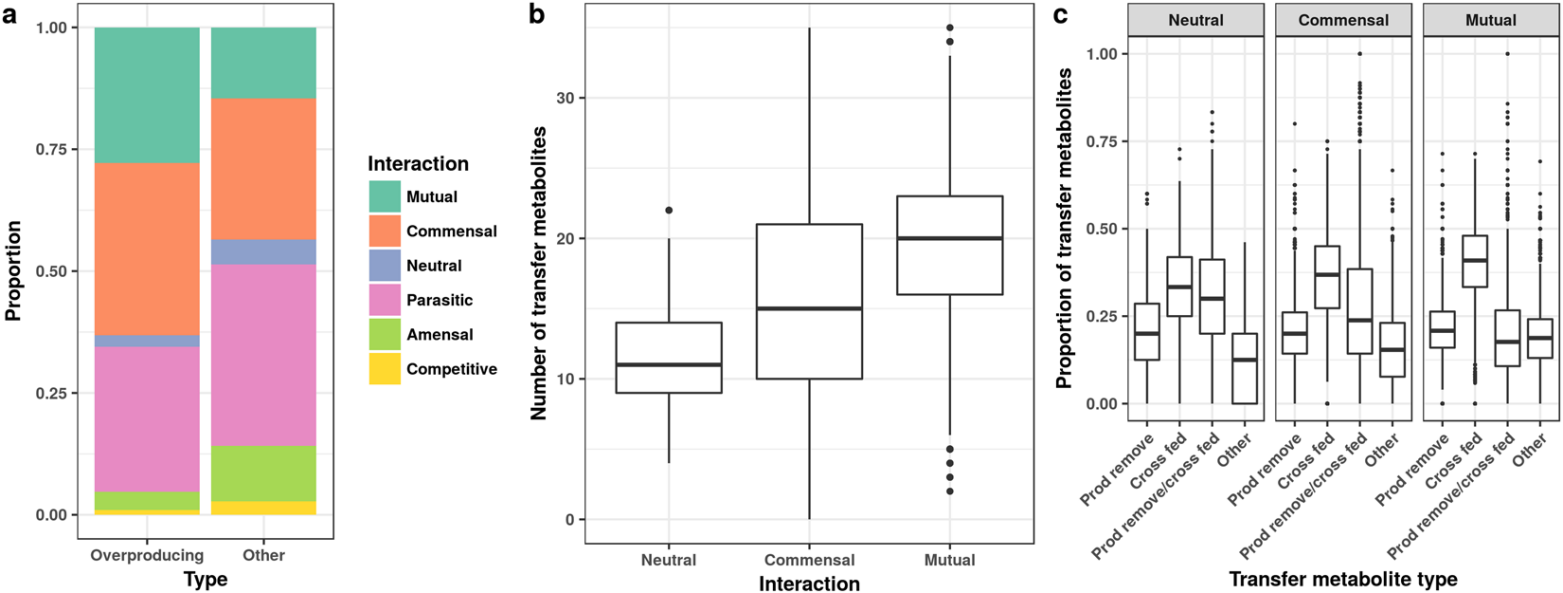
Overproducing co-cultures are enriched for mutualistic and commensal interactions. For all pairwise model simulations with an anaerobic Western diet, ecological interaction types were assigned as described in Methods. (**a**) The proportion of interaction types were plotted for all pairwise combinations (Overall, 297549 combinations) and for combinations that produced a positive exchange flux greater than 10% more than the additive monoculture fluxes (Overproducing, 12125 combinations). (**b**) For overproducing combinations, the total number of transfer metabolites was compared between interaction types. (**c**) For overproducing combinations, each transfer metabolite was classified as: “Cross fed” if taken up by the microbe in monoculture and co-culture simulations, “End product removal” if output for one monoculture and input for the other microbe in co-culture, “End product removal/cross fed” if both classifications applied, and “Other” if neither classification applied.

We next asked why these co-cultures have fluxes that are greater than the sum of the parts. From comparisons of interaction types, overproducing co-cultures were enriched in mutualistic and commensal interactions as compared to the distribution of interaction types amongst all co-cultures (Fig. 4a, Fisher’s exact test, mutualistic: *p* < 2.2e-16; commensal *p* < 2.2e-16; neutral, parasitic, amensal, competitive *p* = 1). We filtered for overproducing co-cultures that were neutral, commensal, or mutualistic because these interaction types are likely to be more long-lived in a dynamic bioreactor scenario. We examined the frequency of transfer metabolites between models in these co-cultures and observed that mutualistic overproducing co-cultures exchanged more metabolites compared to neutral or commensal co-cultures (Fig. 4b, ANOVA *p* =< 2.2e-16, F-value = 810.9). These transferred metabolites fall into different transfer types: “End product removal”; “Cross fed”, “End product removal and cross fed”; and “Other” (see Methods). We observed that “Cross fed” metabolites are enriched in overproducing co-cultures, particularly with mutualistic interactions (Fig. 4c). We further probed which metabolites are most frequently transferred for each transfer classification (Fig. S2), and observed that “Cross fed” metabolites include most amino acids, whereas “End product removal” metabolites include many SCFAs (Fig. S2). These results indicate that strategies to increase chemical production would benefit by developing mutualistic interactions with many cross fed metabolites, including SCFAs and amino acids.

### Co-culturing modifies internal networks via combinations of transfer metabolites that correlate with hub precursor metabolites

Based on the extracellular exchanges outlined above, we queried how these metabolites influence internal pathways thus resulting in modified outputs. We hypothesized that these changes would likely manifest in changes to hub metabolite network statistics. Therefore, we created networks for each organism (see Methods). To find hub metabolites, we calculated the mean degree and betweenness centrality for each metabolite over all monocultures and defined hubs as having the highest values of each metric (Fig. S3). Twelve of the thirteen precursor metabolites described in Noor *et al*.^33^ were identified as hubs. Since these metabolites represent branch points in metabolism for energy generation and synthesis of biomass components (nucleic acids, lipids, amino acids, cell wall), we decided to focus on these “hub precursors” for downstream analyses.

**Figure 5:**
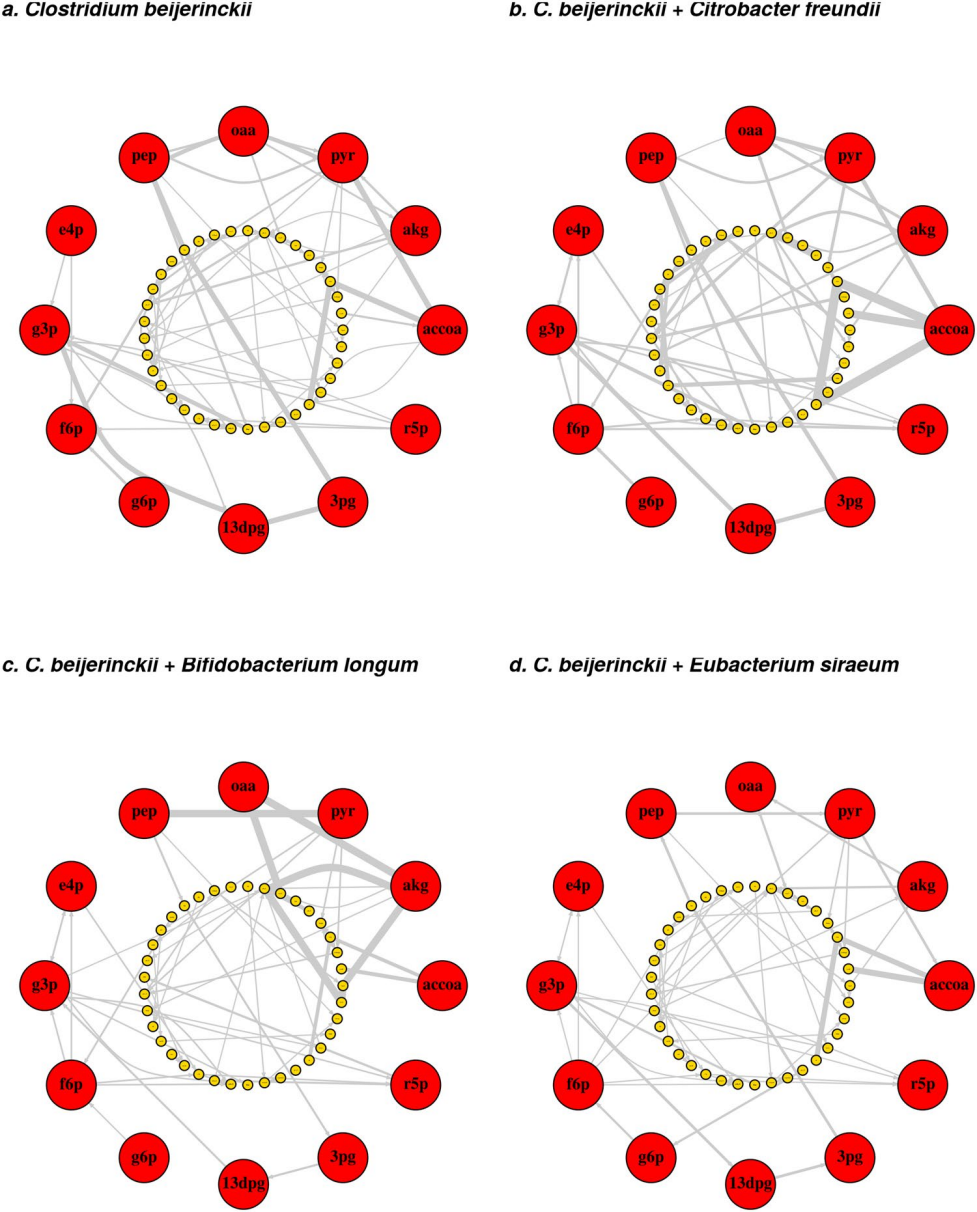
Co-culturing differentially modifies internal network structure for *Clostridium beijerinckii* NCIMB 8052. The network for *C*. *beijerinckii* NCIMB 8052 was created as described in Methods. After monoculture (**a**) and co-culture simulations with a Western diet (**b**–**d**), the networks for *C*. *beijerinckii* were pruned according to reactions in the strongest connected component with absolute flux greater than 1e-05 mmol/gDCW/h and plotted with *igraph*^49^. To compare plots, the common set of metabolite nodes was circle plotted with circles indicating metabolites and edges indicating reactant to product connections. Line width indicates log_2_(flux + 1). Hub precursor metabolites were plotted in the outer circle in red to emphasize flux changes. Metabolite abbreviations are included in Supplementary Information.

To further examine the effects of co-cultures on specific metabolic network phenotypes, we extracted the pairwise simulations for *Clostridium beijerinckii* NCIMB 8052. This microbe is predicted to produce hydrogen gas in monoculture, and some co-cultures result in greater than additive production. To uncover the internal metabolic shifts that result in these phenotypes, we analyzed macroscopic network flux shifts for *C*. *beijerinckii* simulated in monoculture (Fig. 5a) or simulated with other models (Fig. 5b–d). Large shifts in the number of connections and edge weights were particularly evident for the hub precursor metabolites (Fig. 5). To quantify the relationships between hub precursor network statistics and metabolic outputs, we used Mantel tests for overall correlation and significance and canonical correspondence analysis (CCA) for visualization. By Mantel tests, we first calculated significant correlations between the distance matrices of metabolic output fluxes by co-culture and transfer input fluxes by co-culture (r = 0.895, p = 0.001) normalized hub precursor betweenness by co-culture (r = 0.361, p = 0.001), and normalized hub precursor degree by co-culture (r = 0.101, p = 0.001).

**Figure 6:**
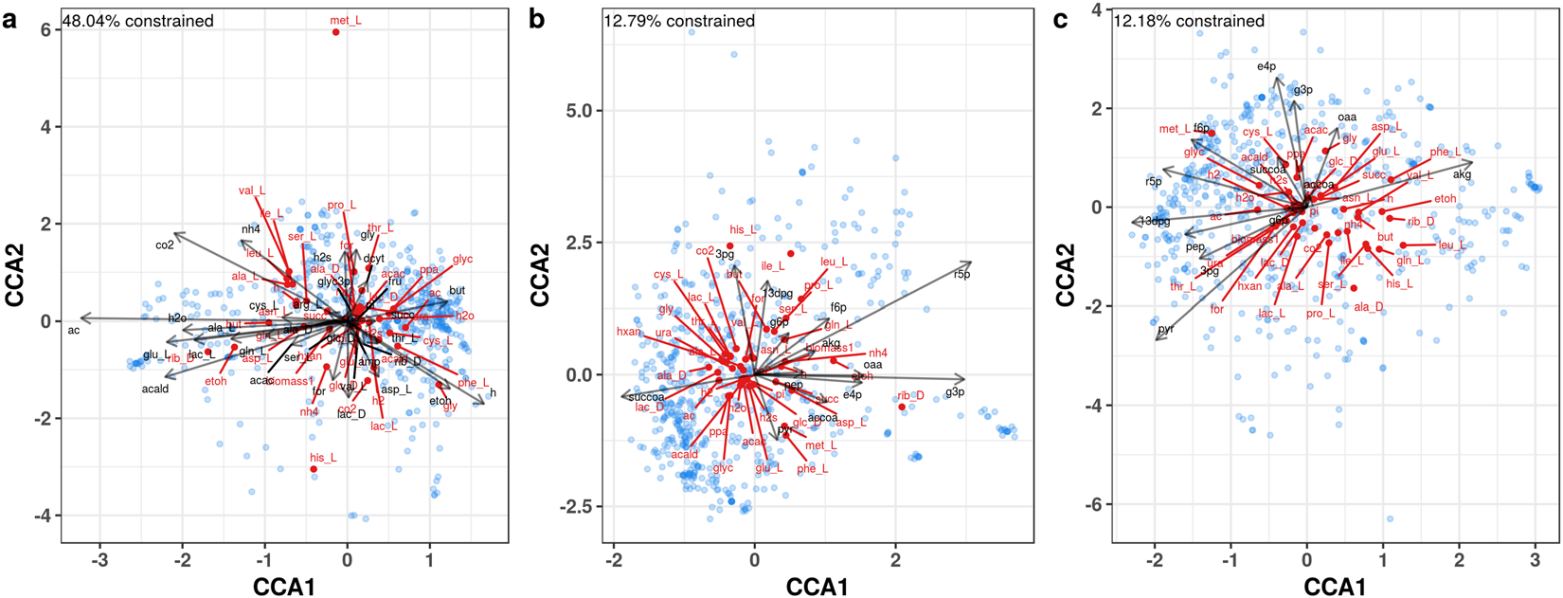
Metabolic outputs from *C*. *beijerinckii* NCIMB 8052 are correlated with combinations of transferred metabolites and hub precursor network statistics. *C*. *beijerinckii* overproduces H_2_ in co-culture. To find metabolic inputs and network statistics that correlate with H_2_ production and other output metabolites, we used canonical correspondence analysis (CCA) to calculate multivariate correlations between combinations of metabolic inputs and network statistics with *C*. *beijerinckii* outputs in co-cultures (anaerobic Western diet). The response matrix of *C*. *beijerinckii* metabolic output fluxes (red points and text) by co-culture (log_10_ + 1 transformed) was used as the community matrix for the *cca* function in *vegan*. The following explanatory matrices (black arrows and text) were separately used to constrain the community matrix: (**a**) transfer metabolite fluxes into *C*. *beijerinckii* (log_10_ + 1 transformed), (**b**) Betweenness centrality of hub precursor metabolites, and (**c**) Degree of hub precursor metabolites. Blue points indicate CCA scores for co-cultures. To limit our analysis to major fluxes, we filtered out input and output fluxes below 1 mmol/gDCW/h. Metabolite abbreviations are included in Supplementary Information.

CCA is an appropriate tool to assess which combinations of hub precursors correlate with the matrix of *C*. *beijerinckii* metabolic output fluxes by co-culture. In addition to hub precursor betweenness centrality and degree, we also constrained output fluxes by transfer fluxes into *C*. *beijerinckii*. The goal of this analysis was to uncover combinations of transfer metabolites that can direct metabolic outputs through paths involving hub precursors. We observed that betweenness and degree constrained 12.79% and 12.18% of outputs, respectively (Fig. 6b,c). Transferred metabolites into *C*. *beijerinckii*, on the other hand, constrained 48.04% of outputs (Fig. 6a), indicating that the other member of the co-culture greatly influenced internal metabolism of *C*. *beijerinckii*. For example, acetate that was transferred into *C*. *beijerinckii* was correlated with butyrate output and butyrate input was correlated with acetate output (Fig. 6a). This finding is supported in the literature in which either acetate or butyrate was supplied to acidogenic monocultures resulting in an increase in production of the other acid^34,35^. Production of H_2_ was correlated with L-threonine, D-ribose, ethanol, and H^+^ inputs (Fig. 6a), succinyl-CoA degree (Fig. 6b), and 1,3-bisphosphoglycerate betweenness (Fig. 6c). These multivariate correlations point to the ability of co-cultures to deliver combinations of inputs that will direct outputs via hub precursor metabolites.

## Discussion

This study leveraged GSMMs derived from the human gut microbiome to predict whether co-cultures can better remediate food waste to commodity chemicals, when compared to single organisms. From comparison of pairwise to single model simulations, we predicted that every microbe model had a higher biomass flux when in co-culture. Furthermore, we identified co-cultures that have the potential to produce greater than additive fluxes for commodity chemical production. These whole greater than the sum co-cultures were enriched for mutualistic and commensal interactions and exchanged more metabolites than neutral co-cultures. Furthermore, these transfer metabolites were most often cross fed rather than removing end products. In the case study of *C*. *beijerinckii*, we observed that the combination of cross-fed metabolites was correlated with internal network structure, thus highlighting the ability of multi-species metabolic interactions to simultaneously modify another member’s phenotype. This study represents how consortia modeling via GSMMs provides a powerful, in silico framework in which to evaluate complex combinatorial interactions to subsequently inform experimentation. Our results predict that pairwise combinations of microbes can more effectively remediate food waste with the added benefit of producing commodity chemicals at greater rates.

Though GSMMs can remarkably predict single and multi-species phenotypes^15,17,18,21^, they are constrained by annotation accuracy and model refinement. Magnusdottir *et al*.^30^ extensively curated 773 AGORA models to approximately the same level as to allow for equal comparison. There are certainly misannotations and incorrect reaction constraints that could only be found by meticulously conducting single isolate experiments, as recently demonstrated in Tramontano *et al*.^36^. The biomass objective is also generalized and we know this to be highly species specific^37^. For multi-species interactions, GSMMs neglect non-metabolic interactions (toxins, nutrient scavenging factors, quorum sensing factors). We thus advocate for the use of GSMMs to screen and identify consortia of interest. These co-cultures then require *in vitro* experimentation to evaluate prediction accuracy and aid in the decision to invoke non-metabolic interactions in further modelling.

The choice of FBA to evaluate combinations of GSMMs is another limitation. We assumed that consortia are at a steady-state with no spatial structure or dynamic interactions, clearly not the case in nature. However, single point FBA allowed us to screen all pairwise combinations of 773 models, which would have taken exponentially more time if considering multiple spatial factors over time. Dynamic FBA (dOptCom^38^, DMMM^18^) and dFBA with spatial components (COMETS^22^, BacArena^19^) could be used to further evaluate greater than additive co-cultures identified in our screen. These methods can further inform experimentation parameters, and provide kinetic frameworks that could also model non-metabolic interactions. The steady-state assumption may convolute predictions of long-term species interactions, especially species with large differences in growth rates^39^. A recently developed FBA method, SteadyCom, provides a framework in which to model consortia with time-averaged constant growth rates of all members, thus providing community stability^39^. Given this point, we filtered our combinations of interest to mutualistic and commensal interactions. These interaction types are more likely to promote long-term multi-species co-existence^40^.

For our combinatorial screen, we used parsimonious FBA (pFBA), which sets the biomass flux to the maximum obtained with FBA, and subsequently minimizes the total absolute flux. The assumption with pFBA is that organisms seek to minimize enzyme production, which has been shown to be an appropriate assumption in recapitulating single organism growth^41^. For evaluating large numbers of combinations, pFBA has the advantage of providing a single solution. Thus, we did not compute the flux range (via FVA) for reactions in co-culture. Though we modeled the same microbe combinations in Magnusdottir *et al*.^30^, the correlation of biomass fluxes was good, but not perfect. Our use of pFBA likely did not bias our findings, since the biomass fluxes are first maximized with FBA. However, we used a different linear solver (GLPK vs. CPLEX) and we did not constrain lower and upper biomass reaction bounds as was done in Magnusdottir *et al*.^30^. Experimental co-cultures have demonstrated that microbes can be completely inhibited by co-culturing, so we chose not to constrain lower biomass bounds above zero. Also in contrast to Magnusdottir *et al*.^30^, we only simulated microbes with a Western and High-Fiber diet under anaerobic conditions (Supplementary Table 1). The gut is an anaerobic environment so we wanted to limit our conclusions to the most natural context. The Western diet better approximates the Army’s nutritional program than the High-Fiber diet in AGORA simulations, so we limited the main results to the Western diet (Figs 1–6). Diet composition will certainly change metabolic inputs, outputs, and transfers for co-cultures, so we repeated the simulations with the High-Fiber diet and observed the same trends predicted by the Western diet simulations (Supplementary Figs 4–12). We cannot guarantee that these trends will hold for all diet compositions, but genome-scale modeling provides an excellent framework to first computationally assess combinatorial feedstock effects on monoculture and consortia metabolisms in such a way that is infeasible *in vitro*.

The microbe combinations identified in this study with the ability to overproduce commodity chemicals represent numerous avenues of future research. From an ecological standpoint, we found an overrepresentation of mutualistic and commensal interactions, and the mutualistic interactions generally involved more cross-fed metabolites (Fig. 4). The mutualism derives from the uptake of toxic end-products from one member to the other, and also cross feeding of biomass building blocks. An ideal pairwise interaction would involve metabolites that are end products for one organism and food the other organism. These predictions provide a framework for choosing microbes to synergistically produce a chemical of interest.

Interestingly, the case study of *C*. *beijerinckii* demonstrated the complex metabolic changes that occur in response to different microbes. The combination of transferred metabolites coordinate to modify the internal network structure. Microbes can metabolically alter outputs from other microbes by transferring metabolites that will exert effects through hub precursor metabolites. From an industrial perspective, these predictions highlight the potential of co-culturing as an alternative to genetic manipulation for modifying and optimizing target chemical production. Typically, industrial fermentations involve highly controlled monoculture conditions with genetic modifications to increase flux through desired metabolic pathways. Co-culturing offers a means to redirect metabolism for microbes that do not have established genetic modification methods, and thus opens the door to microbes with unique metabolisms for industrial commodity chemical production.

This work represents the first step in designing agile and efficient consortia. These simulations now provide a framework to understand multi-species interactions after bringing these isolates into the laboratory for experimentation. The study also demonstrates the power of the human gut microbiota in a new context, food waste remediation to commodity chemicals. We also envision this approach for the design of probiotic combinations of microbes to direct a set of prebiotic nutrients to desired SCFAs, for example. Note that consortia can also overproduce detrimental metabolites such as TMAO (Fig. 3a), which has been linked to heart disease^42^. This approach also scales up to test the effects of more complex consortia (3+), but obviously the computations increase exponentially. Overall, this study predicts that consortia can outproduce monocultures. The benefits of consortia have been observed in several case studies^13,43^, but we predict that by selectively choosing different combinations of microbes, we can direct the same food waste nutrients to different useful products.

## Methods

### Single genome scale metabolic model simulations

Genome-scale metabolic models for 773 gut microbes (AGORA v 1.01) were downloaded from http://vmh.uni.lu ^30^. As noted on their website, models were modified after publication^30^. These modifications included gap-filling for 17 models that could not grow under anaerobic conditions, replacement of unlikely reversible reactions with irreversible reactions, and changes to exchange flux bounds for diet components. The lower and upper exchange bounds for the Western and High-Fiber diets are included in Supplementary Table S1.

All simulations and analyses were performed in R v3.4.0^44^ with plots created using *ggplot2* v 2.2.1^45^. All custom scripts are included in Supplementary Information. Briefly, SBML models were uploaded with *sybilSBML* v 3.0.1^46^ package, and the *sybil* v2.0.4^32^ package was used for model manipulations and FBA. Exchange reaction bounds were modified according to Supplementary Table S1 and growth was simulated with FBA to maximize biomass flux using the GLPK solver (*glpkAPI* package v 1.3.0^47^). The maximum biomass flux was then used to run parsimonious enzyme usage FBA (pFBA, mtf algorithm in *sybil*) so that the total absolute flux was minimized. All resulting single model biomass fluxes are included in Supplementary Table S2. For downstream analyses, only simulations with biomass flux greater than 0.001 h^−1^ were used.

### Pairwise GSMM simulations

Growth for all pairwise combinations of the 773 models were simulated using the Western diet described above. For each combination, models were combined in a similar manner to Magnusdottir *et al*.^30^. The COBRA^31^ MATLAB script, createMultipleSpeciesModel.m found at: https://github.com/opencobra/cobratoolbox, which is based on the FBA implementation in Klitgord and Segre^26^, was used as a template. This script was converted to work in R with the *sybil* package (Supplementary Information) and creates a common environment for metabolites to be exchanged between models, but does not create a host compartment as in the COBRA implementation. After combining the models, exchange fluxes were updated for the Western diet, and pFBA was used to simulate growth, simultaneously maximizing each model’s growth and minimizing the total absolute flux. In contrast to Magnusdottir *et al*.^30^, the lower biomass flux bound was set to 0 and the upper bound set to 1000. All simulations were not able to be solved (given a 5 min timeout) resulting in solutions for 297549 of 298378 combinations. All pairwise biomass fluxes are included in Supplementary Table S3. For downstream analyses, only co-culture simulations that resulted in biomass fluxes of both models greater than 0.001 h^−1^ were used.

Ecological interaction types were assigned as in Magnusdottir *et al*.^30^. If the co-culture biomass flux was at least 10% greater than the monoculture biomass flux, a positive effect was assigned (+1). If the co-culture biomass flux was at least 10% less than the monoculture biomass flux, a negative effect was assigned (−1). Otherwise, a neutral effect was assigned (0). The following interaction types were then assigned: (0, 0) neutral; (0, 1 or 1, 0) commensal; (1, 1) mutualistic; (−1, −1) competitive; (0, −1 or −1, 0) amensal; (−1, 1 or 1, −1) parasitic. Transferred metabolites were classified as follows: “Cross fed” if taken up by the microbe in monoculture and co-culture simulations, “End product removal” if output for one monoculture and input for the other microbe in co-culture, “End product removal and cross fed” if both classifications applied, and “Other” if neither classification applied.

### Network analysis

To create network graphs for each GSMM, species specific reactions and any reactions involving external metabolites were removed. These reactions included those with the following labels: “Transport, extracellular”, “Exchange/demand reaction”, “Exchange”, “Fatty acid synthesis”, “Cell wall biosynthesis”, “Nucleotide interconversion”, “Terpenoid backbone biosynthesis”, “Lipopolysaccharide biosynthesis”, and “Glycerophospholipid metabolism”. For each remaining reaction, edges were created for each reactant to product and product to reactant if the reaction is reversible. Highly connected metabolites were then removed, including H^+^, H_2_O, ATP, ADP, AMP, NAD^+^, NADH, NADP^+^, NADPH, Pi, PPi, CoA, O_2_, NH_4_^+^, H_2_O_2_, SO_4_^2−^, SO_3_^2−^, NO_2_^−^, NO_3_^−^, H_2_S, H_2_CO_3_, NO, S, Fe^2+^, Fe^3+^, Co^2+^, HCO_3_^−^, and H_2_. Note that metabolite abbreviations are included in Supplementary Information. Currency metabolite pairs were also removed according to Hamilton *et al*.^48^. Graphs were further pruned according to simulated FBA fluxes. Networks were pruned to the strongest connected component and any edges corresponding to a reaction with absolute flux less than 1e-05 mmol/gDCW/h were removed. The *igraph* package v 1.1.2^49^ was used to construct the networks and calculate normalized degree and betweenness for each metabolite.

### Statistical analyses

To test the relationship between biomass fluxes calculated in this study and those calculated in Magnusdottir *et al*.^30^, Pearson’s product-moment correlation (cor.test(method = “pearson”, alternative = “two.sided”) function in R) was used to test the alternative hypothesis that the true correlation was not equal to zero. To test the overrepresentation of ecological interaction types in overproducing co-cultures vs. all co-cultures, Fisher’s exact test (fisher.test(alternative = “greater”) function in R) was used on the contingency table of interaction types. To test the significance of the differences in number of transfer metabolites in co-cultures by interaction type, ANOVA was used (aov() function in R). To find metabolic inputs and network statistics that correlate with a specific microbe’s output metabolites in co-cultures, we used canonical correspondence analysis (CCA) to calculate multivariate correlations between combinations of metabolic inputs and network statistics with outputs in co-cultures. The response matrix of metabolic output fluxes by co-culture (log_10_ + 1 transformed for fluxes >1 mmol/gDCW/h) was used as the community matrix for the cca() function in *vegan*. The following explanatory matrices were separately used to constrain the community matrix: transfer metabolite fluxes into the model (log_10_ + 1 transformed for fluxes >1 mmol/gDCW/h), normalized betweenness centrality of hub precursor metabolites, and normalized degree of hub precursor metabolites. To emphasize correlations in CCA plots, a scaling factor of four was used for arrows. Mantel tests were used to test the correlation of distance matrices of output metabolite fluxes (log_10_ + 1 transformed) by co-culture with distance matrices of transfer input fluxes (log_10_ + 1 transformed) by co-culture, normalized betweenness centrality of hub precursor metabolites centrality by co-culture, and normalized degree of hub precursor metabolites by co-culture (mantel(method = “spearman”) function in *vegan* with Euclidean distance matrices calculated with dist() function in R). Significance was assessed by 999 permutations.

## Electronic supplementary material

Supplementary information

Code

Data

Dataset 1

Dataset 2

Dataset 3

Dataset 4

## Data Availability

All data generated or analyzed during this study are included in this published article (and its supplementary information files).
