# Supplementary material for: Human gut microbe co-cultures have greater potential than monocultures for food waste remediation to commodity chemicals
Source: Sci Rep. 2018 Oct 22;8:15594. doi: 10.1038/s41598-018-33733-z (PMC6197241; doi:10.1038/s41598-018-33733-z)
Supplement: Supplementary file 1 — Supplementary information [file 41598_2018_33733_MOESM1_ESM.pdf]

Human gut microbe co-cultures have greater potential than monocultures for food waste remediation to commodity chemicals

Authors:

*Matthew A. Perisin, PhD – US Army Research Laboratory, RDRL-SEE-B, 2800 Powder Mill Road, Adelphi, MD 20783, matthew.a.perisin.civ@mail.mil

Christian Sund, PhD – US Army Research Laboratory, RDRL-SEE-B, 2800 Powder Mill Road, Adelphi, MD 20783, christian.j.sund.civ@mail.mil

Supplementary figure legends

Fig S1: Comparison of monoculture and co-culture biomass fluxes for simulations performed in sybil with parsimonious FBA and simulations performed in Magnusdottir et al. 2016.

Biomass fluxes (anaerobic Western diet) were determined for monocultures and co-cultures according to Methods. Fluxes were plotted against fluxes determined in Magnusdottir et al. 2016 for monocultures (a), co-culture member 1 (b), and co-culture member 2 (c). Red lines indicate 1:1 relationships.

Fig S2: Most frequently transferred metabolites.

For each transfer classification listed in Figure 4c, metabolites were counted among all overproducing co-culture simulations (anaerobic Western diet, 12125 total) and plotted most to least frequent. Metabolites were excluded if only present in 5% or less of co-culture simulations. Metabolite abbreviations are included in Supplementary Information.

Fig S3: Monoculture network analysis confirms key central metabolites as hubs.

Monoculture networks were constructed as indicated in Methods. After monoculture simulations with an anaerobic Western diet, networks were pruned according to reactions in the strongest connected component with absolute flux greater than 1e-05 mmol/gDW/h. Normalized degree and betweenness centrality were calculated for each metabolite in each network. The mean betweenness and mean degree were calculated over all monocultures and plotted above. Red points and text indicate the precursor metabolites described in Noor et al. 2010. Green points and text indicate metabolites that are one reaction away from the precursors. Blue points and text indicate all other metabolites. Metabolite abbreviations are included in Supplementary Information.

Fig S4: Co-culture FBA simulations predict combinations that increase biomass flux compared to monoculture.

For each GSMM (773 total bars along the x-axis), FBA was used to simulate steady-state growth (anaerobic High-Fiber diet) of all pairwise combinations of models. The co-culture biomass fluxes were compared to the monoculture biomass flux for each model and deemed positive if the flux was at least 10% greater, negative if the flux was at least 10% less, and neutral otherwise.

Fig S5: Pairwise simulations predict greater than additive commodity metabolic fluxes.

For all monoculture simulations with an anaerobic High-Fiber diet, exchange metabolite fluxes were compared to pairwise additions of monoculture fluxes, and to all pairwise co-culture simulations. Commodity metabolites include butanol (a), methane (b), formaldehyde (c), propionate (d), hydrogen gas (e), and urea (f). All flux units are mmol/gDW/h.

Fig S6: Pairwise simulations predict emergent metabolite fluxes.

For all monoculture simulations with an anaerobic High-Fiber diet, exchange metabolite fluxes were compared to pairwise additions of monoculture fluxes, and to all pairwise co-culture simulations. Emergent metabolites include trimethylamine N-oxide (a) and nitrous oxide (b). All flux units are mmol/gDW/h.

Fig S7: Overproducing co-cultures are enriched for mutualistic and commensal interactions.

For all pairwise model simulations with an anaerobic High-Fiber diet, ecological interaction types were assigned as described in Methods. (a) The proportion of interaction types were plotted for all pairwise

combinations (Overall, 297549 combinations) and for combinations that produced a positive exchange flux greater than 10% more than the additive monoculture fluxes (Overproducing, 10857 combinations). (b) For overproducing combinations, the total number of transfer metabolites was compared between interaction types. (c) For overproducing combinations, each transfer metabolite was classified as: "Cross fed" if taken up by the microbe in monoculture and co-culture simulations, "End product removal" if output for one monoculture and input for the other microbe in co-culture, "End product removal/cross fed" if both classifications applied, and "Other" if neither classification applied.

Fig S8: Co-culturing differentially modifies internal network structure for *Clostridium beijerinckii* NCIMB 8052.

The network for *C. beijerinckii* NCIMB 8052 was created as described in Methods. After monoculture (a) and co-culture simulations with an anaerobic High-Fiber diet (b-d), the networks for *C. beijerinckii* were pruned according to reactions in the strongest connected component with absolute flux greater than 1e-05 mmol/gDW/h and plotted with *igraph*. To compare plots, the common set of metabolite nodes was circle plotted with circles indicating metabolites and edges indicating reactant to product connections. Line width indicates $\log_2$(flux + 1). Hub precursor metabolites were plotted in the outer circle in red to emphasize flux changes. Metabolite abbreviations are included in Supplementary Information.

Fig S9: Metabolic outputs from *C. beijerinckii* NCIMB 8052 are correlated with combinations of transferred metabolites and hub precursor network statistics.

C. beijerinckii overproduces H_2 in co-culture. To find metabolic inputs and network statistics that correlate with H_2 production and other output metabolites, we used canonical correspondence analysis (CCA) to calculate multivariate correlations between combinations of metabolic inputs and network statistics with *C. beijerinckii* outputs in co-cultures (anaerobic High-Fiber diet). The response matrix of *C.*

beijerinckii metabolic output fluxes (red points and text) by co-culture ($\log_{10}$ + 1 transformed) was used as the community matrix for the *cca* function in *vegan*. The following explanatory matrices (black arrows and text) were separately used to constrain the community matrix: (a) transfer metabolite fluxes into *C. beijerinckii* ($\log_{10}$ + 1 transformed), (b) Betweenness centrality of hub precursor metabolites, and (c) Degree of hub precursor metabolites. Blue points indicate CCA scores for co-cultures. To limit our analysis to major fluxes, we filtered out input and output fluxes below 1 mmol/gDW/h. Metabolite abbreviations are included in Supplementary Information.

Fig S10: Comparison of monoculture and co-culture biomass fluxes for simulations performed in sybil with parsimonious FBA and simulations performed in Magnusdottir et al. 2016.

Biomass fluxes (anaerobic High-Fiber diet) were determined for monocultures and co-cultures according to Methods. Fluxes were plotted against fluxes determined in Magnusdottir et al. 2016 for monocultures (a), co-culture member 1 (b), and co-culture member 2 (c). Red lines indicate 1:1 relationships.

Fig S11: Most frequently transferred metabolites.

For each transfer classification listed in Figure S7c, metabolites were counted among all overproducing co-culture simulations (anaerobic High-Fiber diet, 10857 total) and plotted most to least frequent. Metabolites were excluded if only present in 5% or less of co-culture simulations. Metabolite abbreviations are included in Supplementary Information.

Fig S12: Monoculture network analysis confirms key central metabolites as hubs.

Monoculture networks were constructed as indicated in Methods. After monoculture simulations with an anaerobic High-Fiber diet, networks were pruned according to reactions in the strongest connected

component with absolute flux greater than 1e-05 mmol/gDW/h. Normalized degree and betweenness centrality were calculated for each metabolite in each network. The mean betweenness and mean degree were calculated over all monocultures and plotted above. Red points and text indicate the precursor metabolites described in Noor et al. 2010. Green points and text indicate metabolites that are one reaction away from the precursors. Blue points and text indicate all other metabolites. Metabolite abbreviations are included in Supplementary Information.

Supplementary data and code README

```
# All AGORA v.1.01 models obtained from: http://vmh.uni.lu

# Data directory
## InputTables directory: tables of model, diet, metabolites, and rxn names that are input into scripts
## Parsed directory: output tables from analyses that also serve as inputs depending on script

# Code directory - Custom script descriptions:

# Run simulations of mono cultures:
## AGORA_singleBacteria_FBA_sybil.R
## Script to run monoculture FBA simulations using AGORA models
## specify Western or High-Fiber diet
## minimizing total flux with pFBA
## save exchange metabolic fluxes and all rxn fluxes in 2 tables

# Run simulations of all pairwise co-cultures:
## AGORA_Consortia_FBA_Single_Iteration_sybil.sh
## bash script to run simulations in parallel
## specify input table of co-culture names
## specify output directory
```

```
## will run simulations for Western and High-Fiber diets
## run the simulation by calling R script
## include 5 min timeout to exclude stalled simulations

# R script called by AGORA_Consortia_FBA_Single_Iteration_sybil.sh:
## AGORA_CoCulture_FBA_Single_Iteration_sybil.R
## run co-culture simulation with input from bash script
## wrapper script for runConsortiaSimAGORA_sybil.R
## inputs consortia member names, Western or High-Fiber diet, output directory

# R script with co-culture simulation functions:
## runConsortiaSimAGORA_sybil.R
## loads Western or High-Fiber diet constraints and updates models
## simulate growth of consortia containing 1 or more genome scale metabolic models
## join consortia member models using function from createMultipleSpeciesModelWSybil.R
## simulate growth of consortia with pFBA
## save exchange and all rxn fluxes into 2 tables
## also split exchange flux outputs into tables for each consortia member

# R script to join genome-scale models: createMultipleSpeciesModelWSybil.R
## code adapted from Magnusdottir et. al., 2016
## joins one or more models with a common compartment for metabolic exchanges
## no host compartment

# R script: Create_NetworkFileFromGSM.R
## script to convert a genome-scale metabolic model into a network with
## metabolites as nodes and rxns as edges

# R script: CalcNetStats.R
```

```
## compute network stats after monoculture and co-culture simulated growth


# Figures generated with the following R scripts:

# FigS1.R
## compare monoculture and co-culture growth rates between the AGORA paper, COBRA, and Sybil

# Fig1.R
## Plot Figure 1: numbers of negative, neutral, and positive growth effects
## single strain cocult biomass compared to monoculture biomass

# Fig2and3.R
## script to compare metabolite fluxes from
## all monocultures
## all monocultures added together in pairwise combns
## all simulated co-cultures

# Fig4A.R
## plot distribution of ecological interactions for overproducing co-cultures

# Fig4BandC.R
## script to plot comparison of transfer metabolites for overproducing co-cultures

# FigS2.R
## script to plot the distribution of transfer metabolites for overproducing co-cultures by transfer type

# FigS3.R
## plot mean betweenness vs mean degree for monoculture metabolites
```

Fig5.R
create network plots for Clostridium beijerinckii in monoculture or when simulated in co-culture

Fig6.R
script to analyze multivariate correlations between metabolic outputs and
network stats of precursor metabolites

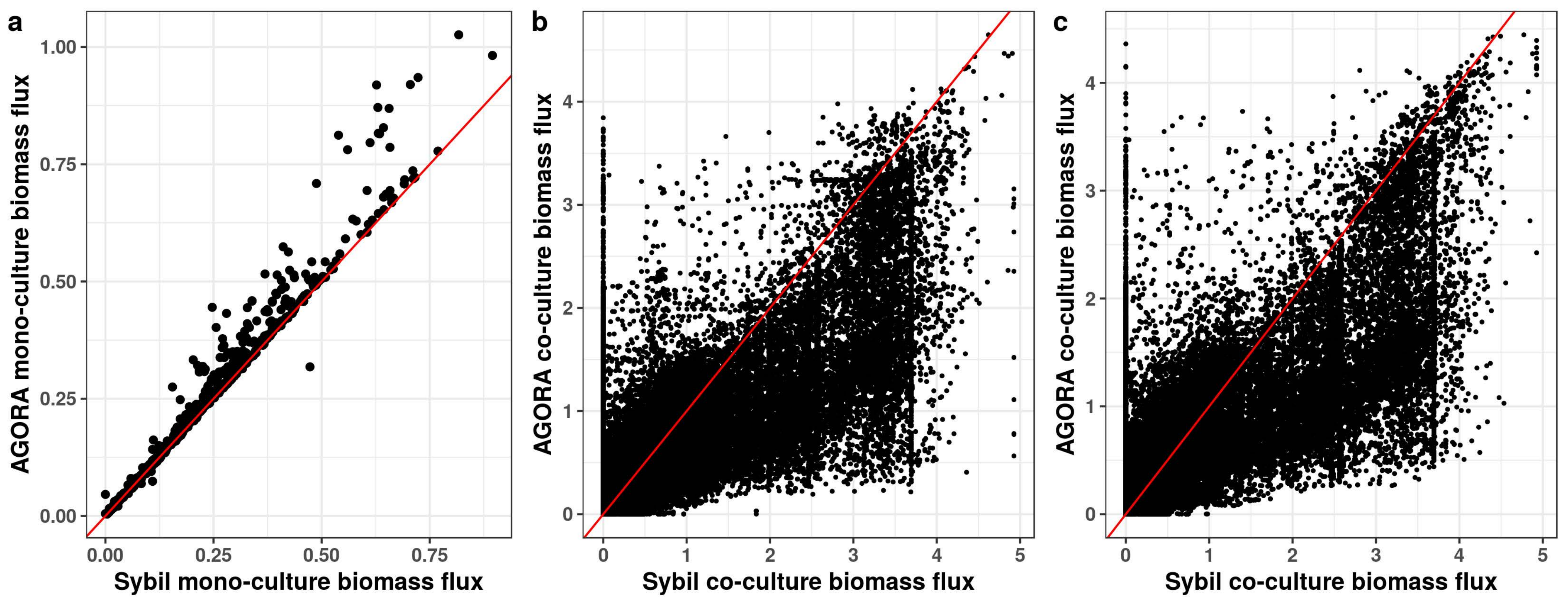

a
b
c
AGORA mono-culture biomass flux
Sybil mono-culture biomass flux
AGORA co-culture biomass flux
Sybil co-culture biomass flux
0.00
0.25
0.50
0.75
1.00
0
1
2
3
4
5

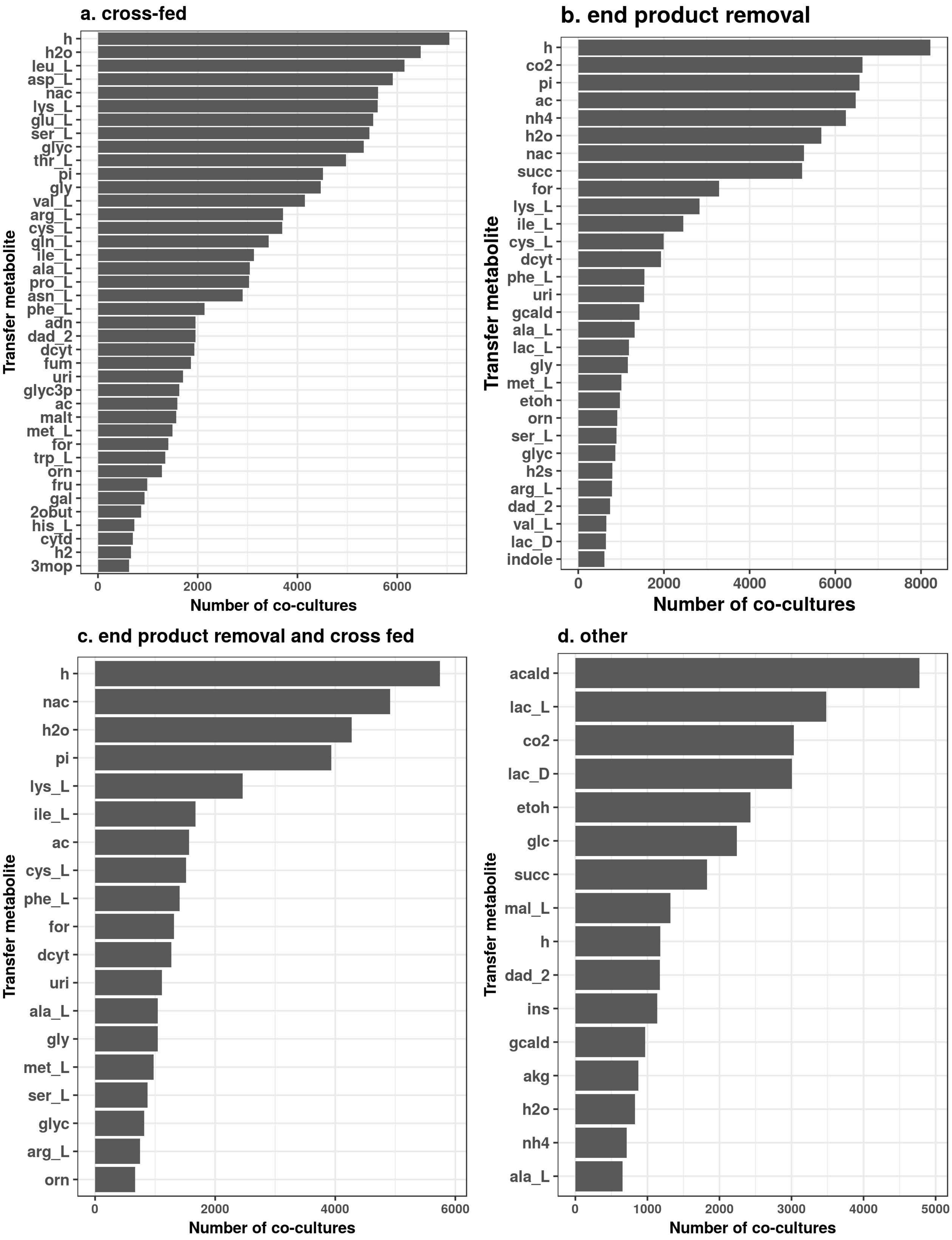
a. cross-fed
b. end product removal
c. end product removal and cross fed
d. other
Transfer metabolite
Number of co-cultures
h
h2o
leu_L
asp_L
nac
lys_L
glu_L
ser_L
glyc
thr_L
pi
gly
val_L
arg_L
cys_L
gln_L
ile_L
ala_L
pro_L
asn_L
phe_L
adn
dad_2
dcyt
fum
uri
glyc3p
ac
malt
met_L
for
trp_L
orn
fru
gal
2obut
his_L
cytd
h2
3mop
co2
nh4
succ
gcald
lac_L
etoh
h2s
lac_D
indole
acald
glc
mal_L
ins
akg
0
1000
2000
3000
4000
5000
6000
8000

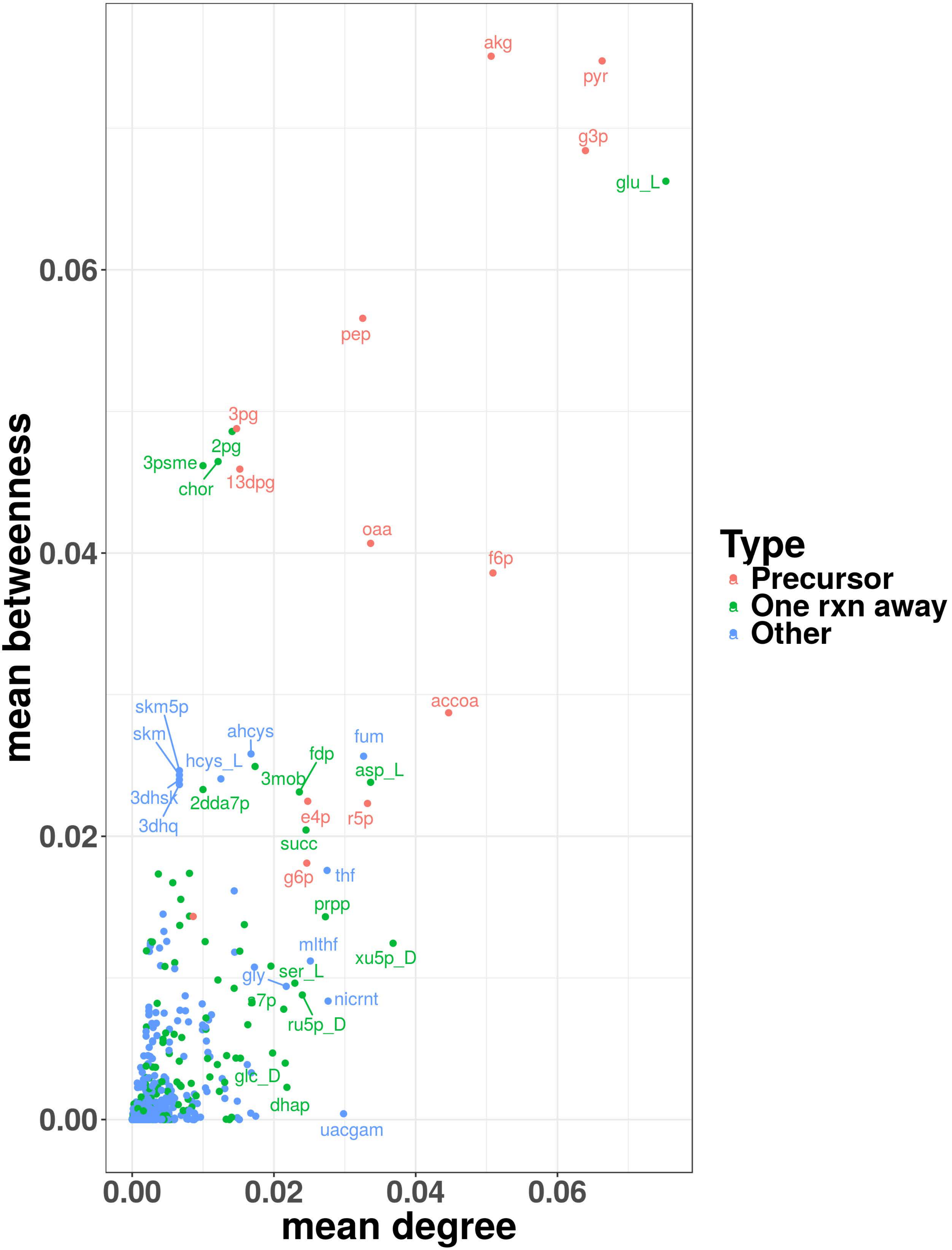

akg
pyr
g3p
glu_L
0.06
pep
3pg
2pg
3psme
chor
13dpg
0.04
oaa
f6p
accoa
skm5p
skm
ahcys
fum
hcys_L
fdp
3mob
asp_L
3dhsk
2dda7p
e4p
r5p
3dhq
0.02
succ
g6p
thf
prpp
mlthf
xu5p_D
ser_L
gly
s7p
nicrnt
ru5p_D
glc_D
dhap
0.00
uacgam
0.00
0.02
0.04
0.06
mean degree
mean betweenness
Type
Precursor
One rxn away
Other

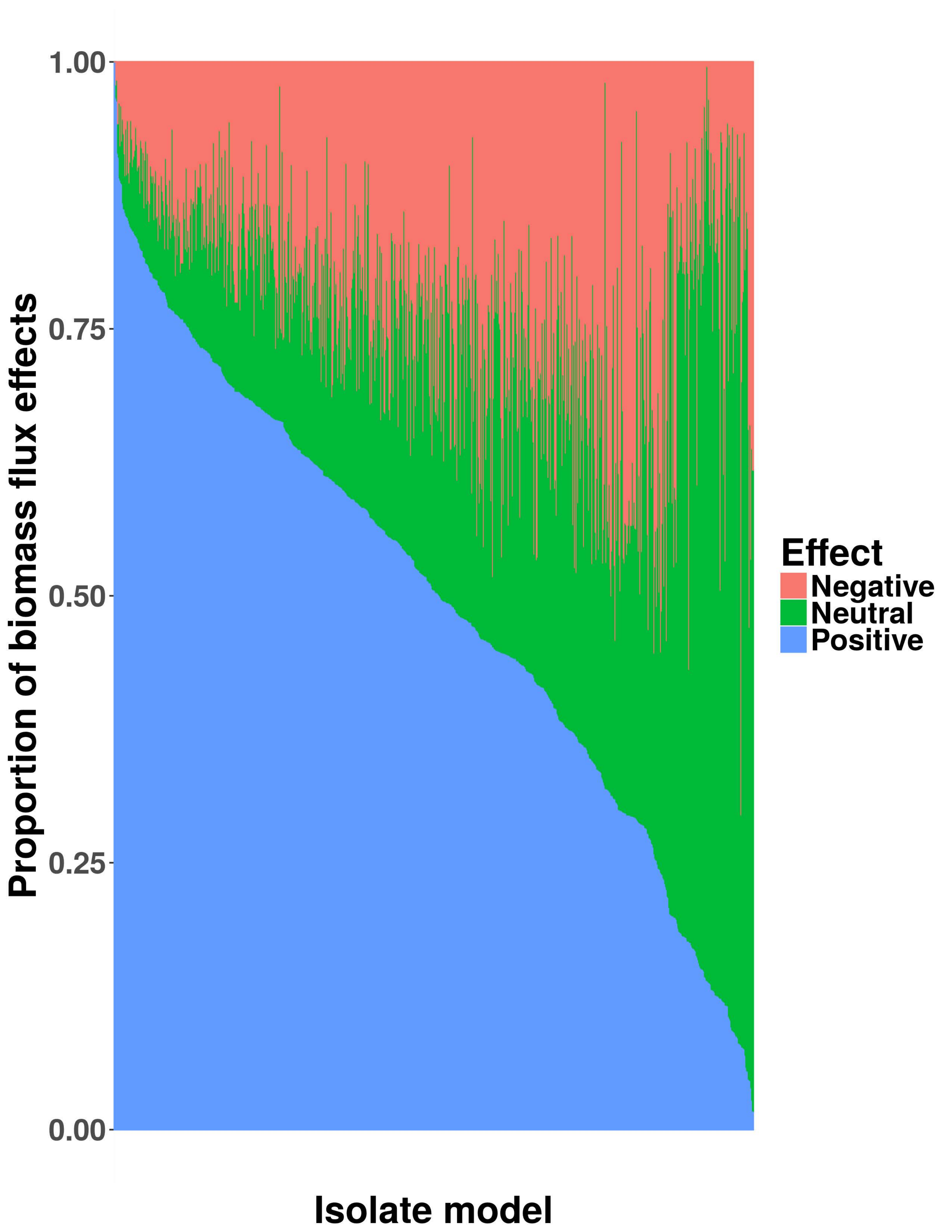

Proportion of biomass flux effects
1.00
0.75
0.50
0.25
0.00
Isolate model
Effect
Negative
Neutral
Positive

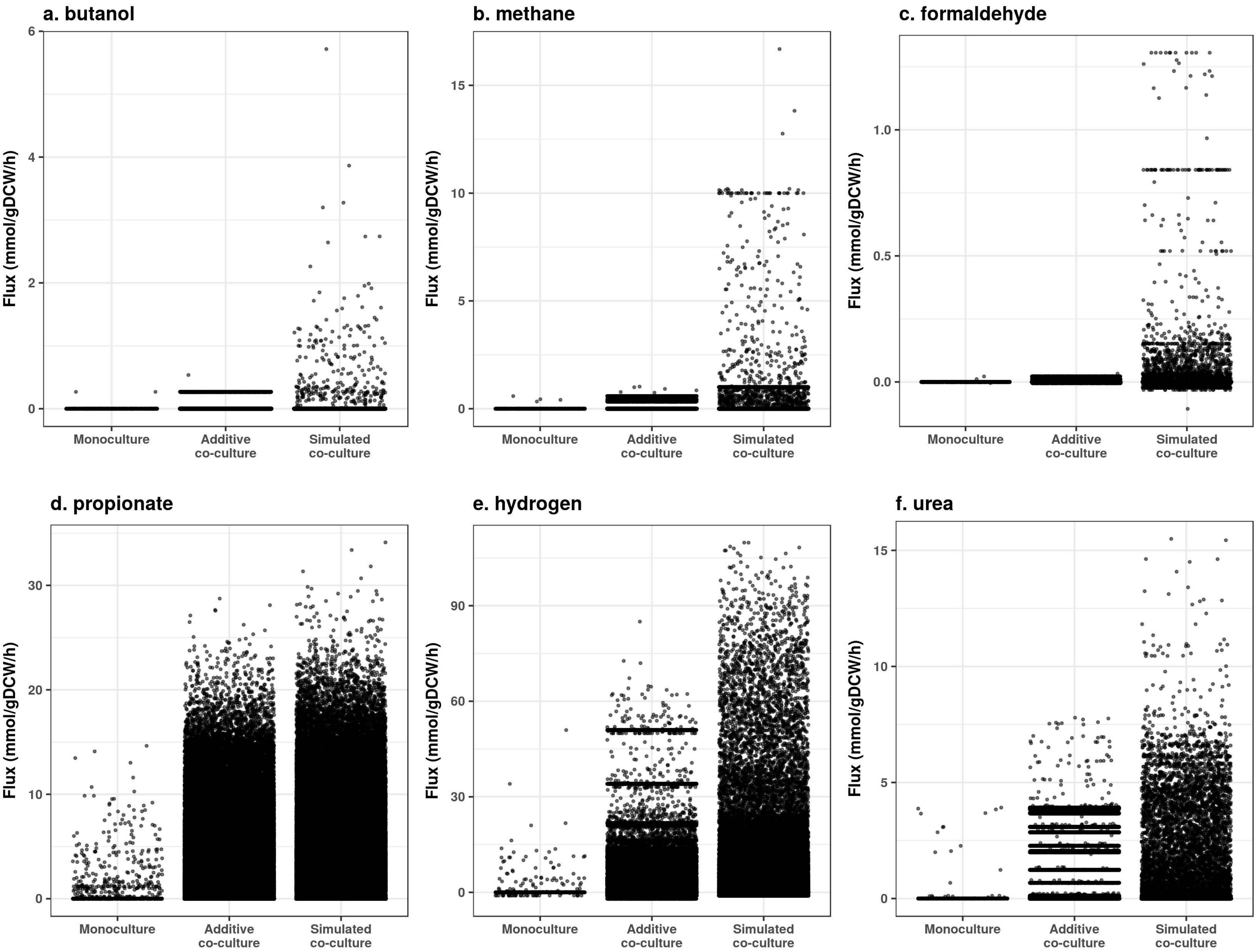

a. butanol
b. methane
c. formaldehyde
d. propionate
e. hydrogen
f. urea
Flux (mmol/gDCW/h)
Monoculture
Additive co-culture
Simulated co-culture
0
2
4
6
5
10
15
0.0
0.5
1.0
20
30
60
90

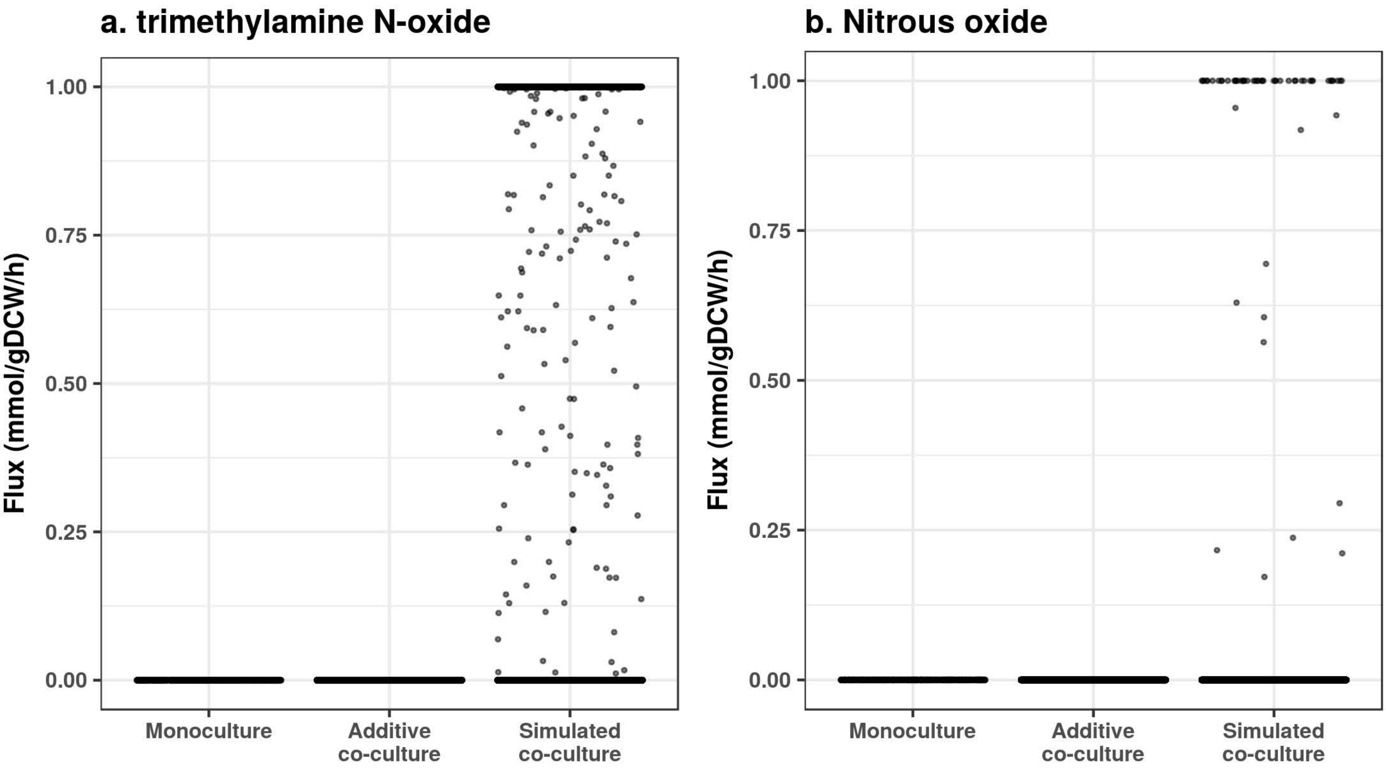

a. trimethylamine N-oxide
b. Nitrous oxide
Flux (mmol/gDCW/h)
1.00
0.75
0.50
0.25
0.00
Monoculture
Additive co-culture
Simulated co-culture

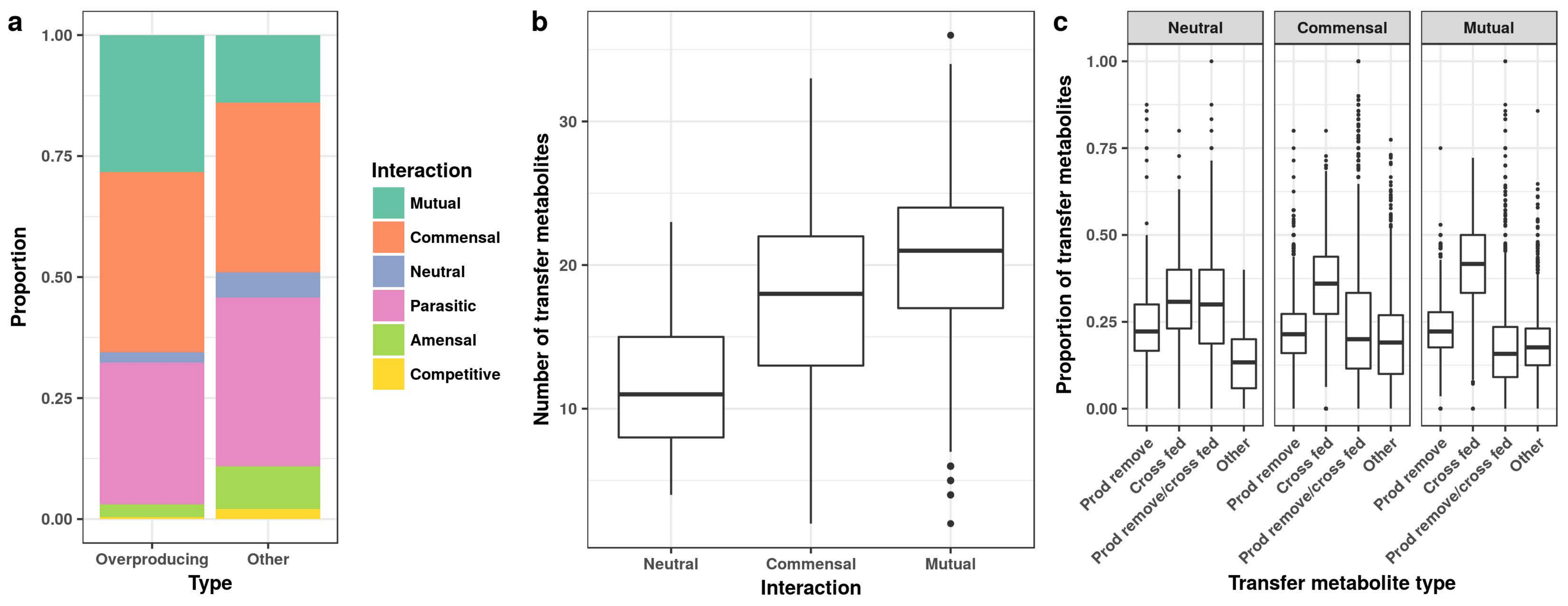

a
1.00
0.75
0.50
0.25
0.00
Proportion
Overproducing
Other
Type
Interaction
Mutual
Commensal
Neutral
Parasitic
Amensal
Competitive
b
30
20
10
Number of transfer metabolites
Neutral
Commensal
Mutual
Interaction
c
Neutral
Commensal
Mutual
1.00
0.75
0.50
0.25
0.00
Proportion of transfer metabolites
Prod remove
Cross fed
Prod remove/cross fed
Other
Transfer metabolite type

a. Clostridium beijerinckii

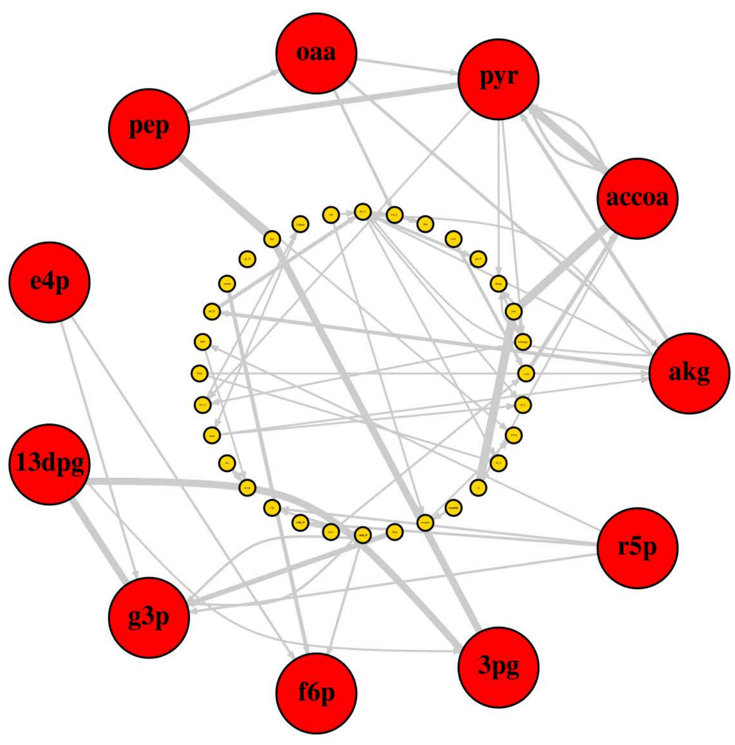

b. C. beijerinckii + Citrobacter freundii

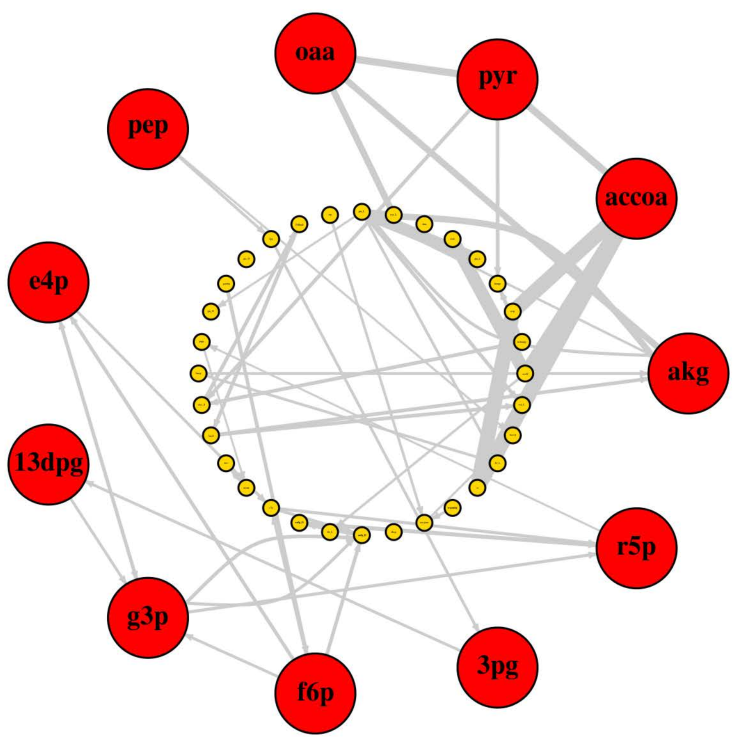

c. C. beijerinckii + Bifidobacterium longum

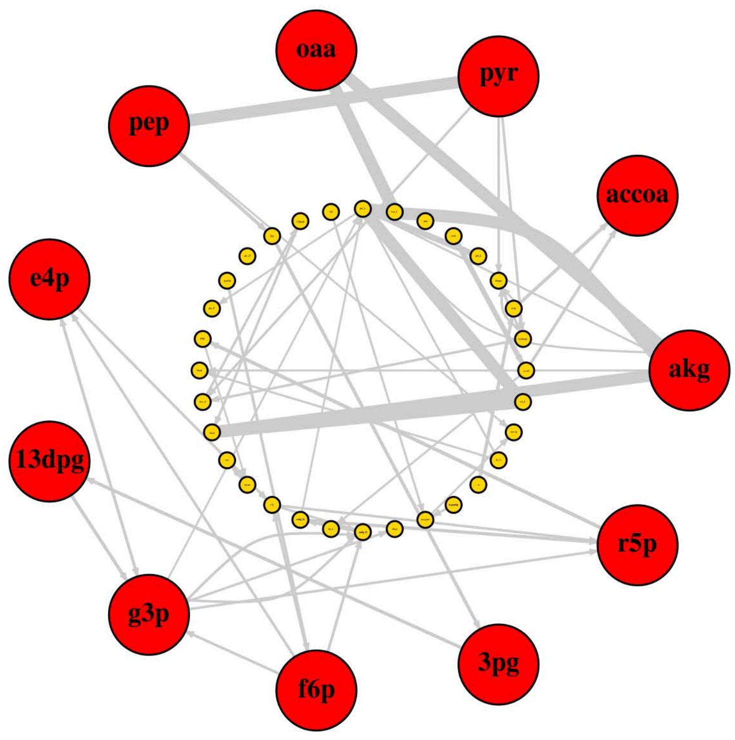

d. C. beijerinckii + Eubacterium siraeum

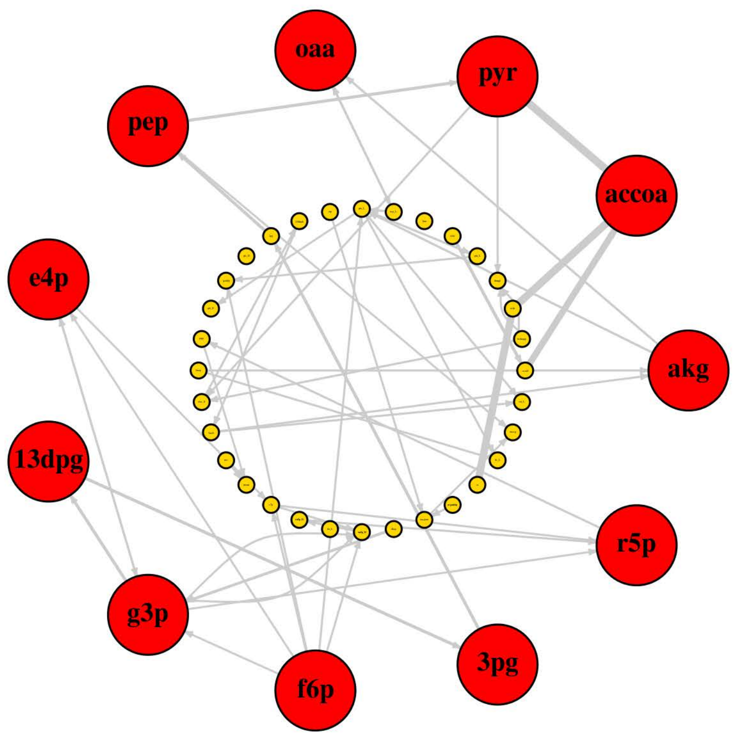

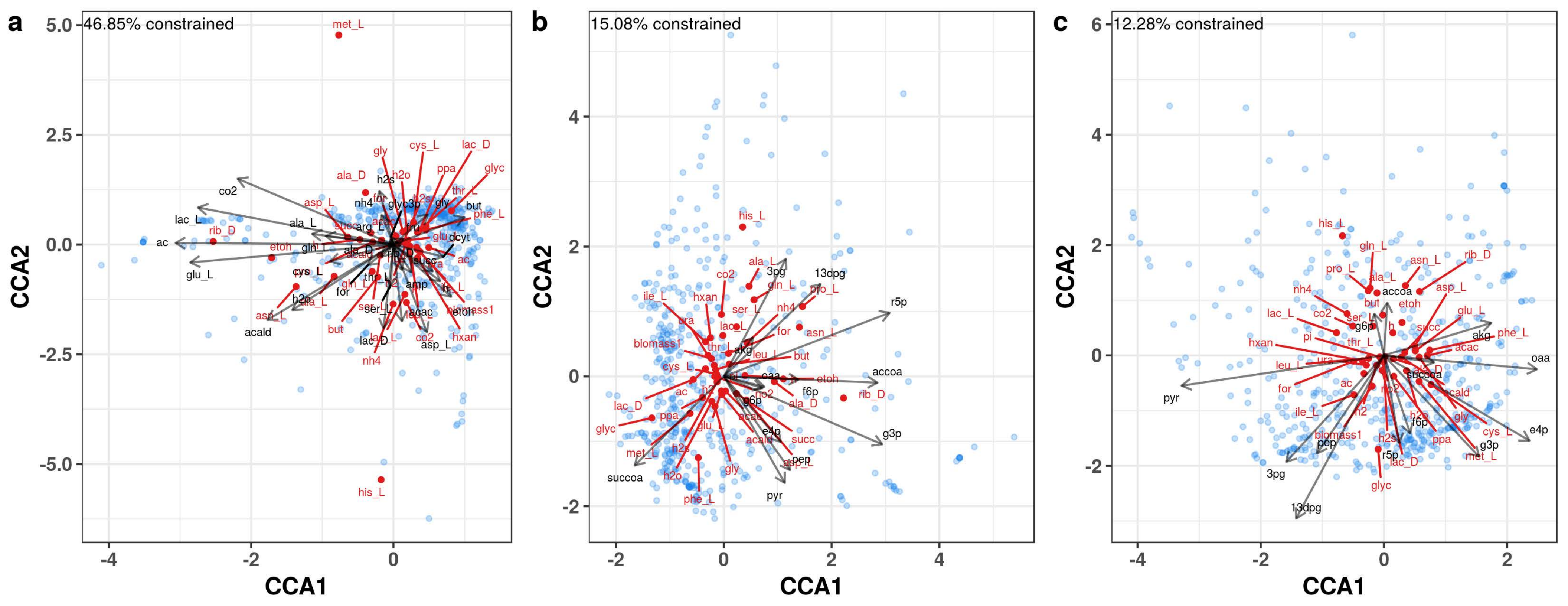
a
46.85% constrained
CCA1
CCA2
b
15.08% constrained
CCA1
CCA2
c
12.28% constrained
CCA1
CCA2

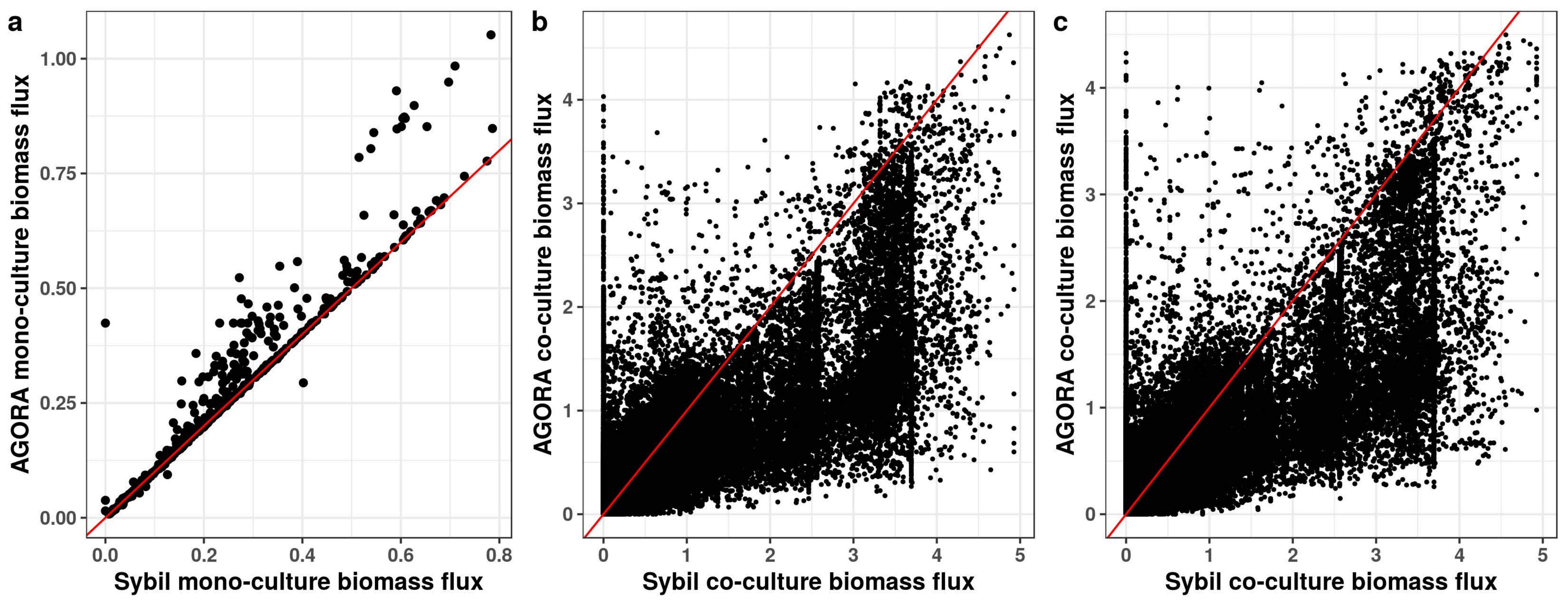

a
b
c
AGORA mono-culture biomass flux
Sybil mono-culture biomass flux
0.00
0.25
0.50
0.75
1.00
0.0
0.2
0.4
0.6
0.8
AGORA co-culture biomass flux
Sybil co-culture biomass flux
0
1
2
3
4
5

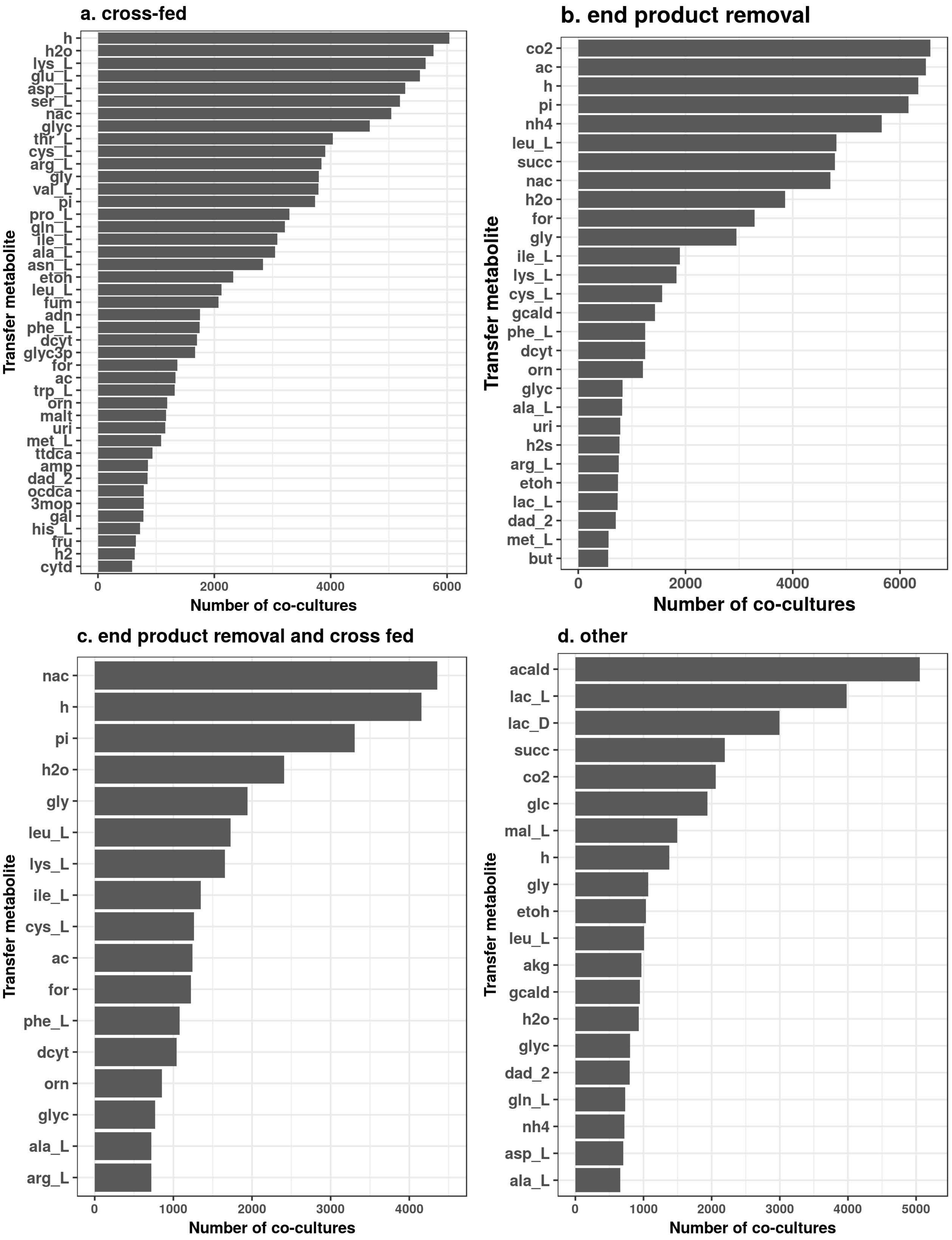
a. cross-fed
h
h2o
lys_L
glu_L
asp_L
ser_L
nac
glyc
thr_L
cys_L
arg_L
gly
val_L
pi
pro_L
gln_L
ile_L
ala_L
asn_L
etoh
leu_L
fum
adn
phe_L
dcyt
glyc3p
for
ac
trp_L
orn
malt
uri
met_L
ttdca
amp
dad_2
ocdca
3mop
gal
his_L
fru
h2
cytd
Transfer metabolite
0
2000
4000
6000
Number of co-cultures
b. end product removal
co2
ac
h
pi
nh4
leu_L
succ
nac
h2o
for
gly
ile_L
lys_L
cys_L
gcald
phe_L
dcyt
orn
glyc
ala_L
uri
h2s
arg_L
etoh
lac_L
dad_2
met_L
but
Transfer metabolite
0
2000
4000
6000
Number of co-cultures
c. end product removal and cross fed
nac
h
pi
h2o
gly
leu_L
lys_L
ile_L
cys_L
ac
for
phe_L
dcyt
orn
glyc
ala_L
arg_L
Transfer metabolite
0
1000
2000
3000
4000
Number of co-cultures
d. other
acald
lac_L
lac_D
succ
co2
glc
mal_L
h
gly
etoh
leu_L
akg
gcald
h2o
glyc
dad_2
gln_L
nh4
asp_L
ala_L
Transfer metabolite
0
1000
2000
3000
4000
5000
Number of co-cultures

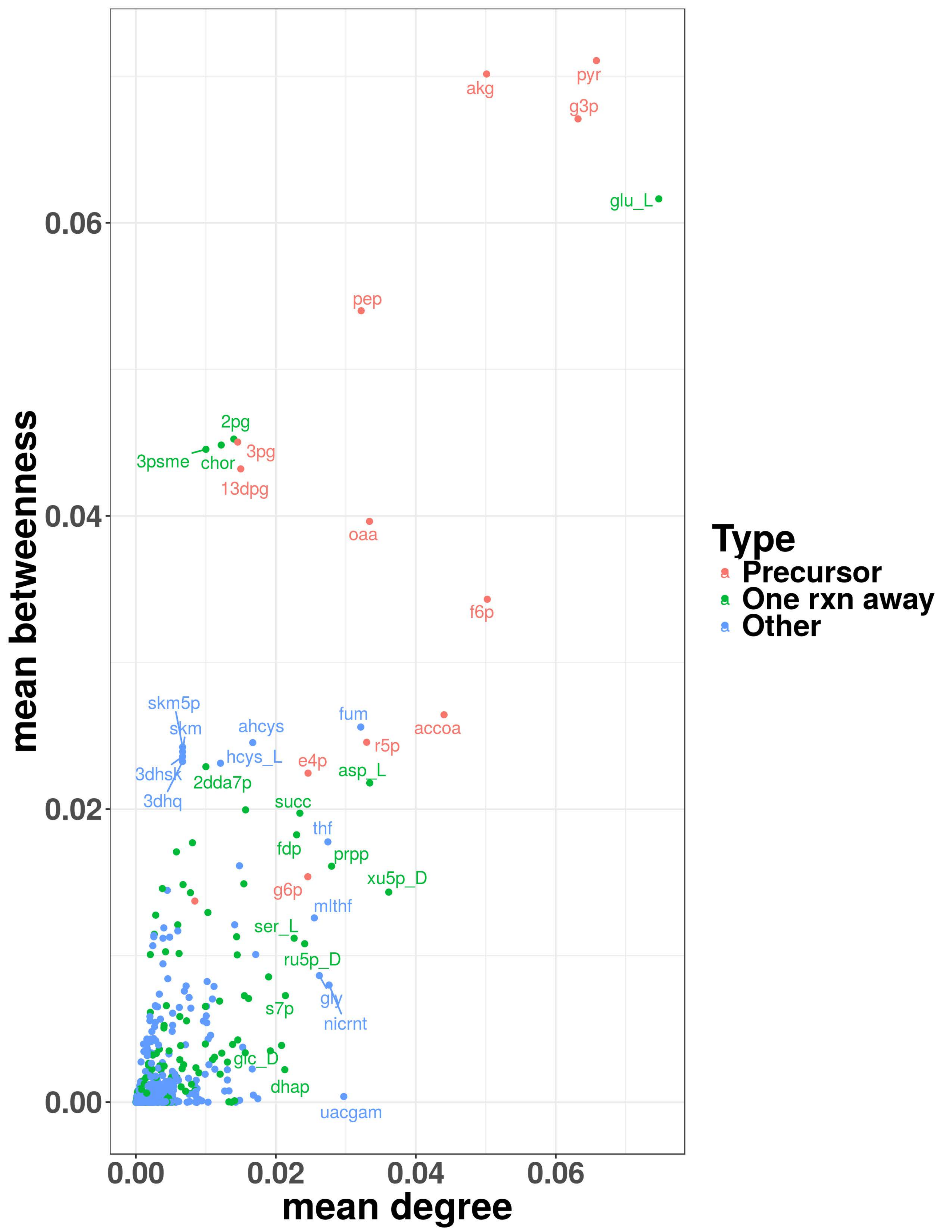

akg
pyr
g3p
glu_L
pep
2pg
3psme
chor
3pg
13dpg
oaa
f6p
skm5p
skm
ahcys
fum
accoa
r5p
hcys_L
e4p
asp_L
3dhsk
2dda7p
3dhq
succ
thf
fdp
prpp
xu5p_D
g6p
mlthf
ser_L
ru5p_D
gly
s7p
nicrnt
glc_D
dhap
uacgam
0.00
0.02
0.04
0.06
mean betweenness
mean degree
Type
Precursor
One rxn away
Other
